# 3D Porous MXene Aerogel through Gas Foaming for Multifunctional Pressure Sensor

**DOI:** 10.34133/2022/9843268

**Published:** 2022-06-27

**Authors:** Yongfa Cheng, Li Li, Zunyu Liu, Shuwen Yan, Feng Cheng, Yang Yue, Shuangfeng Jia, Jianbo Wang, Yihua Gao, Luying Li

**Affiliations:** ^1^ Wuhan National Laboratory for Optoelectronics (WNLO), Huazhong University of Science and Technology (HUST), Luoyu Road 1037, Wuhan 430074, China; ^2^ Information Materials and Intelligent Sensing Laboratory of Anhui Province, Key Laboratory of Structure and Functional Regulation of Hybrid Materials of Ministry of Education, Institutes of Physical Science and Information Technology, Anhui University, Hefei 230601, China; ^3^ School of Physics and Technology, Center for Electron Microscopy, MOE Key Laboratory of Artificial Micro- and Nano-Structures and the Institute for Advanced Studies, Wuhan University, Wuhan 430072, China

## Abstract

The development of smart wearable electronic devices puts forward higher requirements for future flexible electronics. The design of highly sensitive and high-performance flexible pressure sensors plays an important role in promoting the development of flexible electronic devices. Recently, MXenes with excellent properties have shown great potential in the field of flexible electronics. However, the easy-stacking inclination of nanomaterials limits the development of their excellent properties and the performance improvement of related pressure sensors. Traditional methods for constructing 3D porous structures have the disadvantages of complexity, long period, and difficulty of scalability. Here, the gas foaming strategy is adopted to rapidly construct 3D porous MXene aerogels. Combining the excellent surface properties of MXenes with the porous structure of aerogel, the prepared MXene aerogels are successfully used in high-performance multifunctional flexible pressure sensors with high sensitivity (306 kPa ^-1^), wide detection range (2.3 Pa to 87.3 kPa), fast response time (35 ms), and ultrastability (>20,000 cycles), as well as self-healing, waterproof, cold-resistant, and heat-resistant capabilities. MXene aerogel pressure sensors show great potential in harsh environment detection, behavior monitoring, equipment recovery, pressure array identification, remote monitoring, and human-computer interaction applications.

## 1. Introduction

Smart wearable electronic devices are the future development trend of flexible electronics [ 1– 8]. Flexible pressure sensors play an important role in wearable devices, flexible electronic skins, disease diagnosis, intelligent robots, and other fields [ 9– 12]. Flexible pressure sensors can be attached to the skin of robot fingers, palms, or implanted in wearable devices. By converting forces on sensitive components into electrical signals, the perception of external forces and the detection of microelectronics can be realized [ 13– 15]. However, the properties of flexible pressure sensors in practical applications, such as sensitivity, linear detection range, and stability, are often limited by the poor performance of traditional materials [ 16– 18]. Therefore, the development and design of nanomaterials with excellent properties as well as high-performance flexible pressure sensors are of great practical significance for promoting the development of IoT and wearable flexible electronic devices [ 19– 23].

Two-dimensional (2D) MXenes have excellent properties such as good hydrophilicity, tunable interlayer spacing, excellent metal conductivity (9880 S cm ^−1^), and abundant surface functional groups, which have been widely used in flexible electronics, electromagnetic shielding, water purification, energy storage, and catalysis [ 24– 31]. The pioneering work of Ma et al. reported a highly sensitive MXene-based flexible piezoresistive sensor (GF of 180.1) with tunable interlayer distance, demonstrating its potential for applications in pressure sensors [ 32]. However, thin-film solids comprised of 2D materials are prone to stacking and aggregation owing to the interlayer van der Waals forces, which are incompatible with the production of diverse and plentiful conductive channels under pressure, limiting their potential for use in high-performance flexible sensors. Therefore, MXenes are often constructed into three-dimensional (3D) porous structures to make them suitable for flexible electronic skins [ 11, 33– 36]. The 3D porous structures prepared by traditional freeze-drying and template methods have the disadvantages of a complicated preparation process and limited-scale preparation, and 3D printing has rigorous requirements for equipment and process [ 37– 40]. Therefore, it is urgent to develop a method for rapid and large-scale preparation of 3D porous aerogels, so as to fully demonstrate the excellent properties of nanomaterials and extend their applications in future flexible electronics.

Inspired by polymer foaming, we propose a strategy for rapid and large-scale preparation of MXene aerogels, namely, gas foaming [ 41]. The 2D layered MXene films can be rapidly converted into 3D porous MXene aerogels by gas foaming. Under external pressure, the porous aerogel would contact each other between layers and generate enough conductive channels, which is suitable for applications concerning pressure sensors. The 3D porous MXene aerogels can fully exert excellent metal conductivity and large specific surface areas of MXene. By intercalating cellulose nanofibers (CNFs) into interlayers of MXene, the gas foaming effect of MXene aerogel can be greatly enhanced. The prepared MXene aerogel is assembled into a high-performance multifunctional flexible pressure sensor, which enables it to cope with extreme temperatures, mechanical damages, underwater, and other complex environments. The obtained sensor also has capabilities of real-time human activity detection, pressure distribution determination, and human-computer interaction, which can meet the omnifarious needs of future flexible electronics.

## 2. Results

### 2.1. 3D MXene Aerogel and Multifunctional Flexible Pressure Sensor

**Figure 1:**
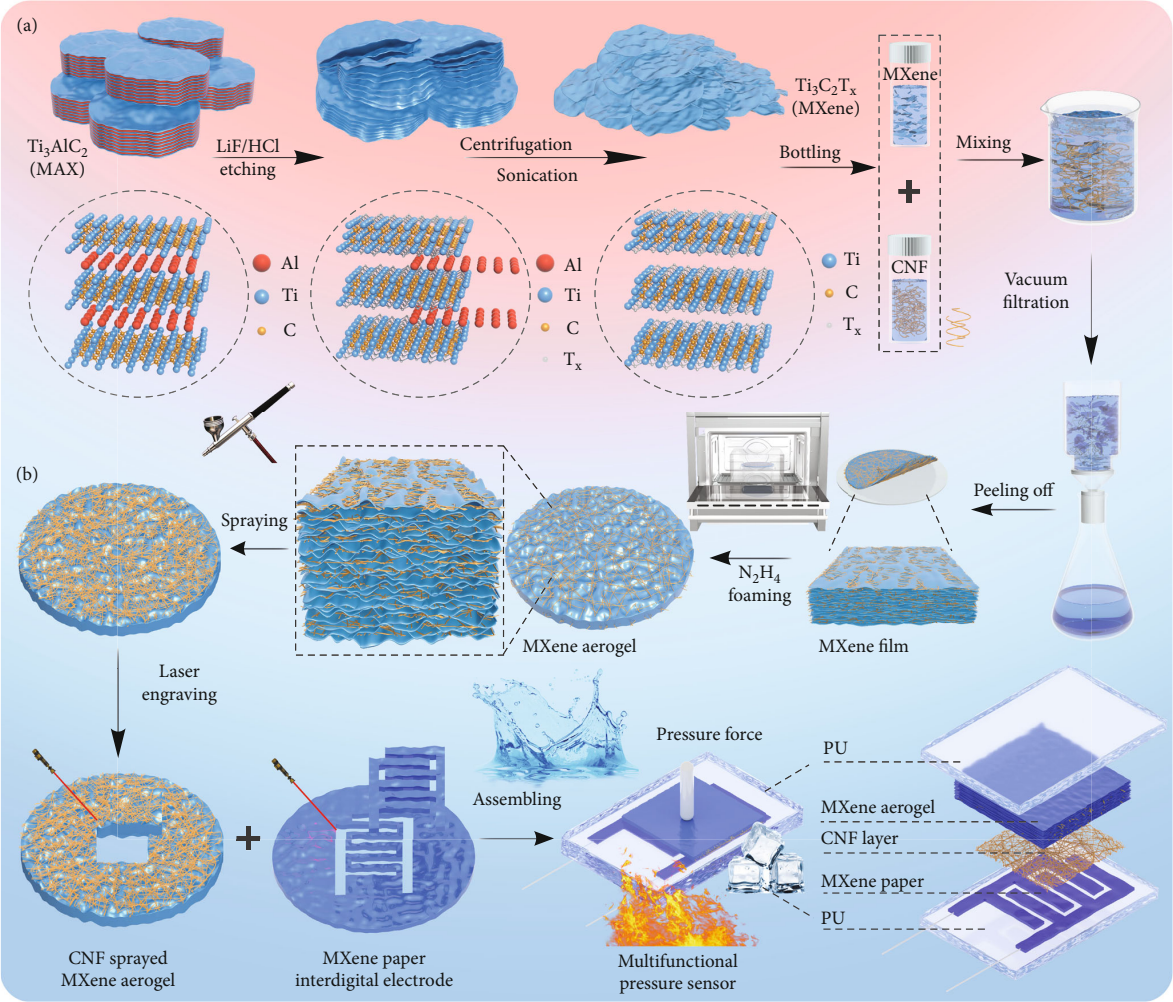
3D MXene aerogel through gas foaming and multifunctional MXene aerogel flexible pressure sensor. (a) The synthesis process of MXene and MXene/CNF composite solution. (b) Schematic illustrations of the evolution of MXene aerogel and the fabrication process of multifunctional MXene aerogel flexible pressure sensor.

First, MXene nanosheets were synthesized by mixing with HCl and LiF following a wet etching process, as shown in Figure 1(a). The prepared MXene nanosheet solution and CNF (Figure S1) were uniformly mixed under magnetic stirring and then dried by vacuum filtration to form MXene/CNF composite films. The prepared MXene/CNF films were placed in a closed environment of hydrazine hydrate (N _2_H _4_) at 90°C (Figure 1(b)), and the resultant 3D porous MXene aerogel can generate enough conductive channels under pressure, which is beneficial to improving the performance of pressure sensors. The isolation layer is also an indispensable part of the pressure sensor, which is often used to improve the sensitivity and pressure detection range of the device. CNFs were sprayed on the surface of MXene aerogel as an isolation layer of the sensor using the thermal spray method [ 33]. MXene paper was then prepared by using a simple method of infiltration in MXene solution, and MXene paper-based interdigital electrodes were customized using the laser engraving technique. Finally, the above components were encapsulated in a PU layer with self-healing properties, and the resultant multifunctional MXene aerogel pressure sensor or sensor arrays (Figure S2) facilitate the detection of pressure at micrometer scale in complex environments without mechanical damage.

### 2.2. Material Characterizations

**Figure 2:**
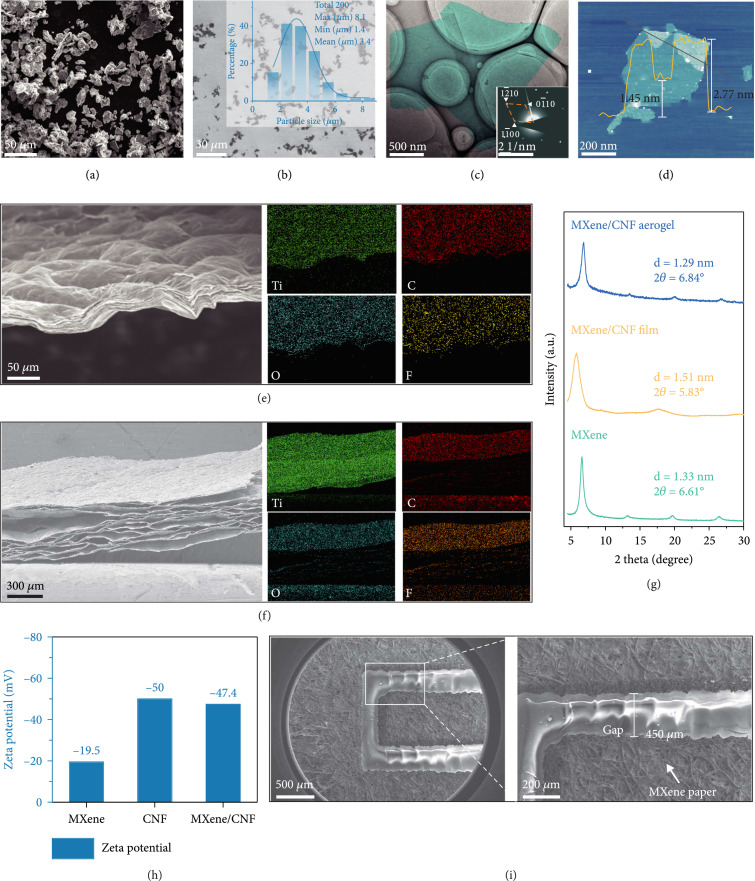
Material characterizations. (a) SEM image of MAX (Ti _3_AlC _2_) phase. (b) SEM image of MXene (Ti _3_C _2_T *_x_*) nanosheets. (c) TEM image of MXene (Ti _3_C _2_T *_x_*) nanosheets and corresponding SAED pattern. (d) AFM image of MXene (Ti _3_C _2_T *_x_*) nanosheets, which shows monolayer thickness of 1.45 nm and bilayer thickness of 2.77 nm. (e) Cross-sectional SEM image of MXene/CNF film and corresponding elemental maps. (f) Cross-sectional SEM image of MXene/CNF aerogel and corresponding elemental maps. (g) XRD spectra of MXene, MXene/CNF film, and MXene/CNF aerogel. (h) Zeta potential of MXene, CNF, and MXene/CNF. (i) SEM image of MXene paper interdigital electrodes (left) and magnified image of the white box region (right).

The key to constructing 3D MXene aerogel pressure sensors lies in the synthesis of high-quality MXene nanosheets. High-purity MAX phase with 500 mesh (Figure 2(a) and Figure S3) was used to ensure sufficient reaction with clean ingredients. After wet etching and multiple centrifugations, the final MXene nanosheet solution took on dark green color with an obvious Tyndall effect (Figure S4). By counting the lateral size of MXene nanosheets in SEM images (Figure 2(b) and Figure S5, S6), the size distribution statistics show that the average lateral size is about 3.4 *μ*m. The TEM image (Figure 2(c)) presents the morphology of a single-layer MXene nanosheet, and its corresponding selected area electron diffraction (SAED) pattern indicates a hexagonal structure of single crystallinity, which is consistent with the reported MXene structure [ 24, 42]. To quantitatively analyze the thickness of synthesized MXene nanosheets, atomic force microscopy (AFM) image (Figure 2(d)) is obtained, which shows that the thickness is either ~1.45 nm (monolayer MXene) or~2.77 nm (bilayer MXene), which proves the successful preparation of single-layer MXene nanosheets. The TEM image (Figure S7) of CNFs shows the morphology of one-dimensional nanofibers. The cross-sectional SEM image (Figure 2(e) and Figure S8) of the MXene/CNF film indicates that it is an obvious layered structure, and the corresponding elemental maps prove uniform distribution of Ti, C, O, and F elements [ 43]. The SEM image of MXene aerogel constructed by the gas foaming technique is shown in Figure 2(f) and Figure S9, which demonstrates a 3D porous interlayer structure, and the elements of Ti, C, O, and F are still uniformly distributed (Figure S10).

To reveal the changes in the interlayer structure of MXene aerogel as compared to MXene film, we performed XRD tests before and after gas foaming. The XRD spectra (Figure 2(g)) show that the (002) characteristic peak of MXene shifts to the left after mixing with CNFs, and that of MXene aerogel after gas foaming shifts to the right. According to the Bragg diffraction law, it can be inferred that the interlayer spacing first increases and then decreases, and the exact values are 1.33 nm, 1.51 nm, and 1.29 nm for MXene, MXene/CNF film, and MXene/CNF aerogels, respectively. The 3D porous MXene aerogel belongs to the microscale morphology, which would not result in the shift of the characteristic peaks. The shift of the (002) peak to the right could be attributed to the depletion of the CNFs. The zeta potentials of MXene, CNF, and MXene/CNF solution (Figure 2(h)) are -19.5 mV, -50 mV, and -47.4 mV, respectively. Since the zeta potentials of both MXene and CNF are negative, they would not agglomerate when mixed. The SEM image and local magnified image of the MXene paper-based interdigitated electrode are shown in Figure 2(i) and Figure S11, S12, S13, and the gap (500 *μ*m in width) between the electrodes ensures that the device would not be short-circuited.

### 2.3. Interlayer Structure and Sensing Mechanism of MXene Aerogels

**Figure 3:**
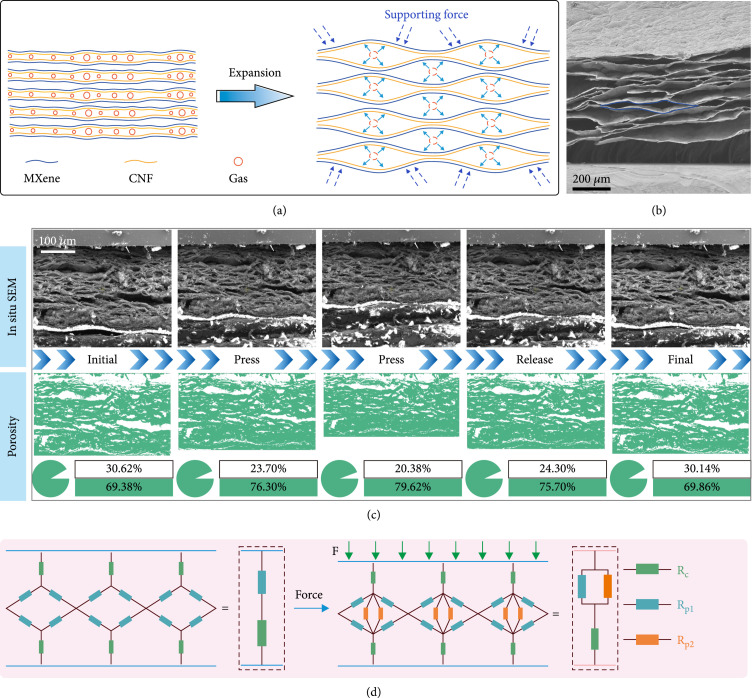
Interlayer structure and sensing mechanism of MXene aerogels. (a) Schematic diagram showing structural changes during gas foaming. (b) SEM image of the interlayer structure of MXene aerogel. (c) In situ mechanical SEM images and corresponding porosity changes during dynamic press and release processes. (d) Equivalent circuit diagram explaining the pressure sensing mechanism.

To reveal the change of the interlayer structure during the gas foaming process and the mechanism for pressure sensing, we further focused on the interlayer changes during the above processes. Figure 3(a) shows the interlayer structural model of MXene. During the process of N _2_H _4_ gas foaming, the interlayer structure of MXene would gradually expand under the inner gas pressure. On the other hand, when the 2D MXene nanosheets are subjected to internal pressure, there would be an interacting force that tries to retain their original shape and prevent the nanosheets from breaking. Finally, a balance would be reached by forming a hyperbolic interlayer structure of the MXene aerogel (Figure 3(b)). To explore the applicability of 3D porous MXene aerogels for pressure sensors, we investigated its pressure sensing mechanism under pressure in the FIB/SEM dual-beam electron microscope (Figure S14). The dynamic in situ mechanical SEM images of the MXene aerogel confirm that it can be effectively compressed and released under pressure (Figure 3(c) and Movie S1). With richer inner contact areas, MXene aerogels show great potential for applications in the field of pressure sensors.

We also calculated the pore area of MXene aerogel to get the real-time porosity in the process of dynamic pressure based on the cross-sectional SEM images (Figure S15 and S16). [ 34] Under the action of pressure, the interlayer porosity of the corresponding aerogel decreases from 30.62% to 20.38%, and after the release, the porosity recovers to 30.14%, which ensures the stable establishment of interlayer conductive channels when the MXene aerogel acts as a pressure sensor. The schematic diagram of the MXene aerogel after gas foaming and the equivalent circuit diagram explaining the sensing mechanism are shown in Figure 3(d). Without the external pressure, the internal resistance of the sensor is composed of contact resistance ( $R_{c}$ ) and inherent interlayer resistance ( $R_{p1}$ ); when pressure is applied, the layers are in contact with each other, which increases the parallel resistance ( $R_{p2}$ ) between interlayers as well as the inherent interlayer resistance, that is, the conductive channels. Therefore, the sensor would receive a dynamic response to the current change. As compared to MXene film, the 3D porous MXene aerogel would expand the variation range of the conductive channels, which can play a significant role in improving the performance of the pressure sensor. [ 44]

### 2.4. High-Performance Multifunctional MXene Aerogel Pressure Sensor

**Figure 4:**
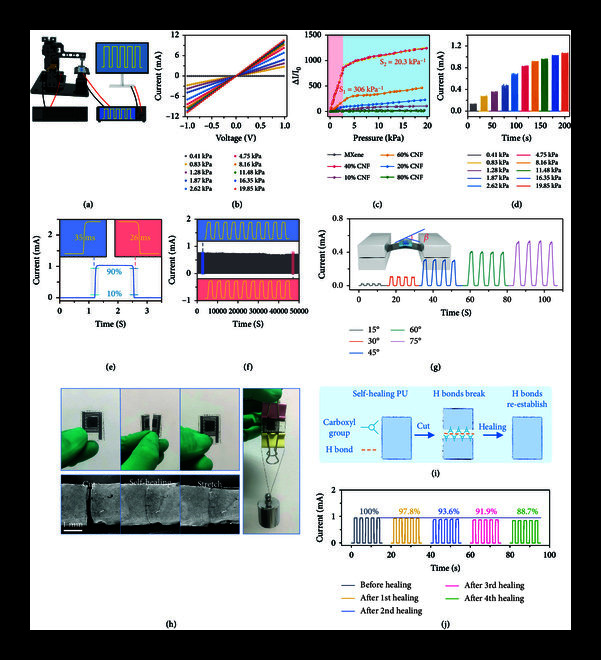
High-performance multifunctional MXene aerogel pressure sensor. (a) Schematic diagram of the high-precision pressure-electrical test system. (b) Ohmic contact characteristics of MXene aerogel sensor. (c) Sensitivity of sensors with different CNF contents. (d) I – T curves of the sensor under various pressures. (e) Response time of the sensor at 16.5 kPa. (f) Cycling stability of the sensor for 20,000 cycles under 3.65 kPa. (g) Tensile properties of the sensor at different bending angles. The inset shows the tensile sensing platform. (h) Self-healing behavior of the sensor (top left), which can bear a weight of 100 g after healing (right), and SEM images showing details of PU self-healing (below left). (i) Self-healing mechanism of PU. (j) Remaining sensing capabilities of the sensor after multiple self-healing treatments at 4.75 kPa.

The MXene aerogel was assembled into a multifunctional flexible pressure sensor using self-healing polyurethane (PU) layers, and corresponding pressure-sensing performance was investigated by placing it in the constructed high-precision motion console (Figure 4(a)). Since the thickness of the isolation layer affects the sensitivity of the sensor, it is determined that 5 mg of CNFs corresponds to the optimal thickness of the isolation layer (Text S2 and Figure S17, S18). A good ohmic contact is always maintained between the MXene aerogel and the electrodes (Figure 4(b) and Figure S19).

The sensitivity ( S ) of a sensor is an important metric for evaluating its performance (Figure 4(c) and Figure S20). The MXene aerogel sensor with 40 wt% CNF shows the highest sensitivity and a wide pressure detection range, exhibiting three linear regions: $S_{1}=306\text{kP}{\text{a}}^{-1}$ within 2.60 kPa, $S_{2}=20.3\text{kP}{\text{a}}^{-1}$ in the range of 2.60 kPa to 20.00 kPa, and $S_{3}=0.81\text{kP}{\text{a}}^{-1}$ in the range of 20.00 kPa to 87.37 kPa (Figure S21). Sensitivity is mainly determined by the changing conductive path, that is the increased contact area between layers under stress (Figure S22). The more the pores, the larger the contact areas under specific pressure. When the pressure is small, many small pores among the layers are in contact with each other, and the sensitivity is high. As the pressure continues to increase, further increase of the contact area would count on the large pores, which requires even greater strain; so, the corresponding sensitivity decreases. The sensor can provide a superior current response to various pressures as compared to other reported sensors (Figure 4(d) and Table S1). The MXene aerogel sensor shows a fast response time (35 ms) and recovery time (26 ms) at 16.5 kPa, as shown in Figure 4(e). The sensor also exhibits outstanding stability during 20,000 pressure cycles at 3.65 kPa (Figure 4(f)). Furthermore, the current response of the sensor can be used to identify the frequency and speed of varying pressures (Figure S21). The capability of sensing tension is an important property of flexible sensors, and this sensor can sensitively detect the bending behavior at different bending angles and speeds (Figure 4(g) and Figure S23).

Additionally, the self-healing PU endows the MXene aerogel sensor with the self-healing property (Figure 4(h) and Figure S24, S25). The sensor self-heals after being cut and can still withstand a weight of 100 g. The SEM images of cut, self-healing, and stretched PU demonstrate the details of the self-healing behavior. The self-healing property is attributed to the large number of hydrogen bond acceptors and donors in the supramolecular network of carboxylated PU, as shown in Figure 4(i). The strong self-healing capability of PU can not only effectively restore the structural integrity of the damaged device but also ensure functional recovery of the device [ 45]. The MXene aerogel sensor can still maintain 88.7% of the sensing ability after the 4th self-healing process at 4.75 kPa, as shown in Figure 4(j). In addition, we prepared 10 batches of MXene aerogel sensors and evaluated their mechanical and sensing properties (Figure S26). The related pressure-strain and current-strain curves exhibit little variation, demonstrating great repeatability and dependability of the MXene aerogel sensors.

### 2.5. Practical Applications of the Multifunctional MXene Aerogel Pressure Sensor

**Figure 5:**
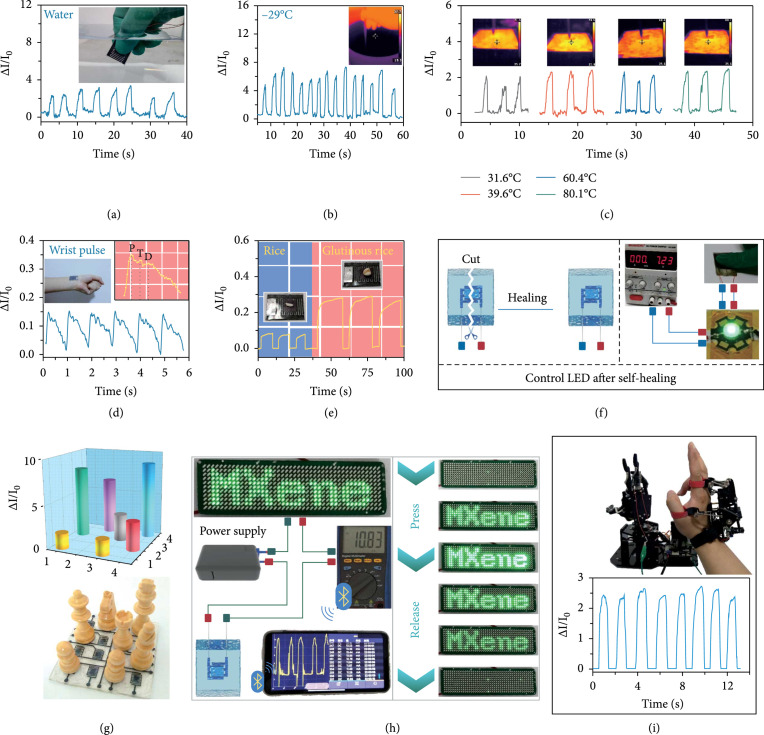
Practical applications of the multifunctional MXene aerogel pressure sensor. (a) Waterproof capability of the sensor. (b) Low-temperature tolerance. (c) High-temperature tolerance. (d) Pulse monitoring capability for medical diagnosis. (e) Ability to detect tiny objects corresponding to a grain of rice (2.3 Pa) and barley (6.0 Pa). (f) Self-healing ability of the sensor, controlling the brightness of an LED after self-healing. (g) The 4×4 MXene aerogel sensor array detects the distribution of chess pieces. (h) Human-computer interaction interface for remote monitoring (left) and controlling the brightness of an LED light sign (right). (i) Use of the sensor in motion monitoring of interactive manipulators.

The MXene aerogel sensor is placed in various harsh environments to explore its environmental applicability. The sensor can still detect the mechanical behavior of finger pressing in water (Figure 5(a)), suggesting that it might be utilized to monitor underwater mechanical signals. Moreover, the sensor is cold and heat resistant and can function properly at temperatures as low as -29.0°C and as high as 31.6°C to 80.1°C (Figures 5(b) and 5(c) and Figure S27).

High-performance multifunctional MXene aerogel pressure sensors have great potential for various practical applications. The sensor is attached to the finger, wrist, and elbow, and during the bending and stretching actions, the sensor can sensitively detect the exact human motion behavior (Figure S28). Real-time monitoring of human pulse signals is an important means of medical diagnosis. When the sensor is attached to the wrist, it can identify the pulse signals (Figure 5(d)). The characteristic peaks of the wrist pulse, such as the “ P ” (percussion), “ T ” (tidal), and “ D ” (dicrotic) waveforms, are sensitively identified. Here, “ P ” and “ T ” correspond to the early and late peaks of systolic blood pressure, and “ D ” reflects the diastolic zone. The radial augmentation index ( T / P ) can be calculated according to the above signals, which is an important parameter to describe the stiffness of the arteries. The sensor can also precisely distinguish the gravity of tiny objects such as a grain of rice (2.3 Pa) and barley (6.0 Pa), as demonstrated in Figure 5(e). Additionally, after the sensor is cut and then self-healed, the brightness of an LED can still be controlled by the self-healed sensor (Figure 5(f)).

We designed a 4×4 MXene aerogel sensor array to explore its capability of sensing pressure distributions (Figure 5(g) and Figure S2). The sensor array can accurately identify the gravity of chess pieces is applicable to detecting small pressure distributions. A human-computer interaction interface for remote monitoring is built using a Bluetooth module (Figure 5(h) and Figure S28). The brightness of the “MXene” sign is controlled by applying pressure to the sensor, and the mobile phone can remotely monitor the state of the circuit in real-time. MXene aerogel sensors can also be used in the sensing and interaction fields of robots. The sensor can recognize the bending behavior of the finger when a human interacts with a robot (Figure 5(i)). In addition, it can also accurately identify the motion behavior of the robot (Figure S28). On the whole, MXene aerogel pressure sensors have great potential in applications including harsh environment detection, behavior monitoring, device recovery, pressure array recognition, remote monitoring, and human-computer interaction.

## 3. Discussion

In conclusion, 2D MXene nanosheets suffer from the disadvantage of easy self-stacking, which limits the development of their excellent properties. The intercalation of CNFs into 2D layered MXenes leads to the formation of 3D porous MXene aerogels by using a fast gas foaming technique. The feasibility and sensing mechanism of MXene aerogel as a pressure sensor was investigated by in situ mechanical SEM at a micrometer scale. MXene aerogel pressure sensor has high sensitivity (306 kPa ^-1^), a fast response time (35 ms), wide pressure detection range (2.3 Pa to 87.3 kPa), ultrastability (>20,000 cycles), and self-healing characteristics, which can achieve various applications in electronic skin, harsh environments, device recovery, remote monitoring, pressure array recognition, and human-computer interaction. The constructed MXene aerogel can exert the excellent surface properties of MXene, realize the combination of nanomaterials and flexible electronics, and show great potential for the development of flexible electronics in the future.

## 4. Experimental

### 4.1. Materials

All chemical reagents were purchased from Aladdin Bio-Chem Technology Co., Ltd. (China) and used without further purification. High-purity Ti _3_AlC _2_ powder (≥400 mesh) was purchased from Laizhou Kai Kai Ceramic Materials Company Ltd. CNF solution was purchased from Guilin Qihong Technology. The water used in all experiments was obtained using a Simplicity Milli-Q system (Millipore, France).

### 4.2. Fabrication of Ti _3_C _2_T *_x_* (MXene) Nanosheets

Firstly, 1 g of LiF powder was added to the prepared 20 ml of 9 M hydrochloric acid. Secondly, 1 g of Ti _3_AlC _2_ powder (MAX phase) was slowly added to the above-mixed solution, followed by continuous magnetic stirring of the mixture at 35°C for 24 h to achieve etching of the Al layer. Thirdly, the reaction product was washed several times with ultrapure water by centrifugation to make its pH value greater than 6. Fourth, the above product was sonicated in an ice bath and argon atmosphere for 1 h. Finally, after centrifugation at 3500 rpm for 30 min, the dark green supernatant of MXene nanosheets was collected.

### 4.3. The Preparation of 3D Porous MXene Aerogels

The prepared MXene and CNF solutions were mixed in different proportions into a series of MXene/CNF solutions (10 wt%, 20 wt%, 40 wt%, 60 wt%, 80 wt% of CNF contents) and then sonicated for 5 min and stirred magnetically for 30 min to make them well mixed. The mass of the MXene films was 30 mg. The specific volume of the mixed solution was poured into the extraction device and finally dried into a series of MXene films. Then, 2 ml of hydrazine hydrate (85 wt%) was added to the MXene films and kept at 90°C for 1 h to achieve gas foaming. MXene film on glass slide in sealed petri dish evolved into MXene aerogels by the vapor of a blowing agent (N _2_H _4_). The MXene aerogels were successfully prepared in this way.

### 4.4. Assembly of the Multifunctional MXene Aerogel Pressure Sensor

Firstly, MXene aerogels were used as the sensitive layer, and CNFs were sprayed on the surface as the isolation layer of the sensor. Secondly, a piece of paper was soaked in the MXene solution, and then MXene paper was prepared after natural drying. After that, MXene paper was engraved into interdigital electrodes by laser engraving technique at the power of 5 W. Finally, a self-healing PU layer was used to assemble the above components into the multifunctional MXene aerogel pressure sensor and sensor array.

### 4.5. Pressure Sensing Mechanism

The sensing mechanism of the sensor is based on the piezoresistive properties of 3D porous MXene aerogel. The resistance of the sensor can be divided into constant resistance and contact resistance between the electrodes and MXene aerogel, forming a resistor network in parallel with the inherent resistance. In this process, the sensitivity of the sensor is determined by the pores changes of the MXene aerogel under external pressure. As an important parameter for evaluating device performance, the sensitivity of the sensor is described as follows:

(1)S=∆I/Ioff∆P,

where ΔI , $I_{\text{off}}$ , and ΔP denote the change of current under pressure, the initial current, and the corresponding pressure change, respectively.

### 4.6. Characterizations

The morphological and structural analysis of MXene nanosheets, CNFs, MXene films, and MXene aerogels were achieved by atomic force microscope (Shimadzu SPM9700), Malvern master size 2000, Raman spectroscopy (Horiba Jobin Yvon Aramis, 532 nm laser), field-emission scanning electron microscope (SEM, FEI NOVA NanoSEM 450), and transmission electron microscope (FEI Titan G ^2^ 60−300). In situ mechanical SEM characterization was realized in a dual-beam SEM/FIB microscope (FEI Quanta 3D FEG) with a nanomanipulator (Oxford Instruments OmniProbe 100). The electrical properties of MXene aerogel sensors were evaluated by a high-precision pressure-electrical test system and the Agilent B2091A Source meter.

## Data Availability

The data used to support the findings of this study are available from the corresponding author upon request.

## Supplementary Materials

Supplementary Materials Text S1: material characterization. Text S2: optimization of the isolation layer of multifunctional MXene aerogel sensor. Figure S1: structure of cellulose nanofibers (CNF, C _6_H _10_O _3_). Figure S2: photographs of MXene aerogel flexible pressure array sensor. Figure S3: SEM images of MAX phase (Ti _3_AlC _2_). Figure S4: Tyndall phenomenon of MXene, CNF, and MXene/CNF solutions. Figure S5: SEM images of Ti _3_C _2_T _X_ MXene nanosheets. Figure S6: SEM images of Ti _3_C _2_T _x_ MXene nanosheets and corresponding statistics of sheet size distribution. Figure S7: TEM image of cellulose nanofibers (CNF, C _6_H _10_O _3_). Figure S8: SEM images of MXene/CNF film. Figure S9: SEM images of MXene/CNF aerogel. Figure S10: cross-sectional SEM images of MXene/CNF film (upper) and aerogel (lower) and the corresponding elemental maps. Figure S11: SEM images of MXene paper interdigital electrodes. Figure S12: (a) Plan-view and (b) cross-sectional SEM images of MXene paper. Figure S13: (a) Plan-view and (b) cross-sectional SEM images and corresponding EDS elemental maps of MXene paper. Figure S14: schematic illustration of the *in situ* mechanical test of MXene aerogel in FIB-SEM. Figure S15: *in situ* SEM images and corresponding porosity changes during dynamic press and release processes. Figure S16: the porosity changes of the MXene aerogel during dynamic press and release processes. Figure S17: SEM images of sprayed CNF isolation layer. Figure S18: sensitivity of MXene aerogel sensor with different thicknesses of CNF isolation layer. Figure S19: ohmic contact characteristics of MXene aerogel sensor. Figure S20: sensitivity of MXene aerogel sensor with different CNF contents. Figure S21: pressure-sensing properties of MXene aerogel sensor. Figure S22: schematic diagram of the change of conduction channel. Figure S23: tension-sensing properties of MXene aerogel sensor. Figure S24: SEM images show the self-healing processes of PU including cut, self-healing, and stretching. Figure S25: photographs of self-healing characteristics of (a) PU and (b) interdigital electrodes. Figure S26: the reliability of MXene aerogel sensors. (a) The mechanical and (b) sensing performances of ten batches of MXene aerogel sensors. Figure S27: the sensor attached to the hand can recognize the grabbing action of a hot water glass. Figure S28: practical applications of the multifunctional MXene aerogel flexible sensor. Figure S29: surface profiles of MXene paper, MXene films, and aerogels with different CNF contents. Figure S30: 3D morphologies of MXene paper, MXene films, and aerogels with different CNF contents. Figure S31: dimensions of interdigital electrodes. Table S1: comparison of 3D MXene aerogel sensor and other sensors. Table S2: surface roughness of MXene paper, MXene films, and MXene aerogels. Movie S1 (mp4) : in situ observation of MXene aerogel during dynamic press and release processes in FIB-SEM. The playing speed is the actual speed.
