# Supplementary material for: 3D Porous MXene Aerogel through Gas Foaming for Multifunctional Pressure Sensor
Source: Research (Wash D C). 2022 Jun 27;2022:9843268. doi: 10.34133/2022/9843268 (PMC11715778; doi:10.34133/2022/9843268)
Supplement: Supplementary Materials — Text S1: material characterization. Text S2: optimization of the isolation layer of multifunctional MXene aerogel sensor. Figure S1: structure of cellulose nanofibers (CNF, C 6H 10O 3). Figure S2: photographs of MXene aerogel flexible pressure array sensor. Figure S3: SEM images of MAX phase (Ti 3AlC 2). Figure S4: Tyndall phenomenon of MXene, CNF, and MXene/CNF solutions. Figure S5: SEM images of Ti 3C 2T X MXene nanosheets. Figure S6: SEM images of Ti 3C 2T x MXene nanosheets and corresponding statistics of sheet size distribution. Figure S7: TEM image of cellulose nanofibers (CNF, C 6H 10O 3). Figure S8: SEM images of MXene/CNF film. Figure S9: SEM images of MXene/CNF aerogel. Figure S10: cross-sectional SEM images of MXene/CNF film (upper) and aerogel (lower) and the corresponding elemental maps. Figure S11: SEM images of MXene paper interdigital electrodes. Figure S12: (a) Plan-view and (b) cross-sectional SEM images of MXene paper. Figure S13: (a) Plan-view and (b) cross-sectional SEM images and corresponding EDS elemental maps of MXene paper. Figure S14: schematic illustration of the in situ mechanical test of MXene aerogel in FIB-SEM. Figure S15: in situ SEM images and corresponding porosity changes during dynamic press and release processes. Figure S16: the porosity changes of the MXene aerogel during dynamic press and release processes. Figure S17: SEM images of sprayed CNF isolation layer. Figure S18: sensitivity of MXene aerogel sensor with different thicknesses of CNF isolation layer. Figure S19: ohmic contact characteristics of MXene aerogel sensor. Figure S20: sensitivity of MXene aerogel sensor with different CNF contents. Figure S21: pressure-sensing properties of MXene aerogel sensor. Figure S22: schematic diagram of the change of conduction channel. Figure S23: tension-sensing properties of MXene aerogel sensor. Figure S24: SEM images show the self-healing processes of PU including cut, self-healing, and stretching. Figure S25: photographs of self [file 9843268.f1.zip › Supplementary Materials.docx]

Supplementary Materials for

**3D Porous MXene Aerogel through Gas Foaming for Multifunctional Pressure Sensor**

Yongfa Cheng^1^, Li Li^1^, Zunyu Liu^1^, Shuwen Yan^1^, Feng Cheng^2^, Yang Yue^2^*, Shuangfeng Jia^3^, Jianbo Wang^3^, Yihua Gao^1^, and Luying Li^1^*

Correspondence to: yueyang@ahu.edu.cn; luying.li@hust.edu.cn

**This PDF file includes:**

Supplementary Texts S1-S2

Figs. S1 to S28

Tables S1 to S2

Movie S1

**Other Supplementary Materials for this manuscript include the following:**

Movie S1 (mp4)

*In-situ* observation of MXene aerogel during dynamic press and release processes in FIB-SEM. The playing speed is the actual speed.

Supplementary Text

**Text S1: Material characterization**

Ti_3_C_2_T_x_ MXene nanosheets are prepared by selective etching of the Al layer in the precursor MAX phase (Ti_3_AlC_2_) using a wet etching process with HCl and LiF. The resultant dark green colloidal dispersion is refined by ultrasonication and centrifugation. The obtained Ti_3_C_2_T_x_ MXene nanosheets are homogeneous in an aqueous solution. MXene and CNF solutions are mixed with good dispersibility, which can be confirmed from the Tyndall phenomenon as shown in **Figure S4**. The TEM image (**Figure S7**) of CNF shows that it has a one-dimensional nanofiber structure.

The plan-view and cross-sectional SEM images of the MXene paper are shown in **Figure S12**. The corresponding EDS elemental maps (**Figure S13**) confirm that MXene is evenly distributed in the MXene paper. The surface profiles of MXene paper, MXene/CNF films, and aerogels indicate that the surfaces are relatively flat (**Figure S27, 28 and Table. S2**). In addition, the self-healing PU has a self-healing ability, which endows the interdigital electrodes with self-healing ability as well (**Figure S23, S24**).

**Text S2: Optimization of the isolation layer of multifunctional MXene aerogel sensor**

The isolation layer is an important intermediate layer to improve the sensing performance of the pressure sensor. The introduction of an isolation layer often leads to improved sensitivity and an extended detection range of pressure sensors. Here, CNF is sprayed to the surface of MXene aerogel as an isolation layer by thermal spraying. The SEM images in **Figure S17A** show that CNFs are evenly sprayed on the Si surface, which confirms the feasibility of the thermal spraying method. Meanwhile, CNFs of different thicknesses are sprayed on the MXene aerogel, as shown in **Figure S17B-D**. The SEM images of 5 mg CNFs sprayed on MXene/CNF foam have an even contrast, while those of 10 mg CNF present brighter contrast due to the existence of a thicker organic layer.

To optimize the thickness of the isolation layer, MXene aerogel sensors with different thicknesses of the isolation layer (2.5 mg, 5 mg, and 10 mg of CNFs sprayed on the foam of 9 cm^2^ in size) are prepared. **Figure S18** shows the sensitivity of the 40wt% CNF MXene aerogel sensor with different thicknesses of the isolation layer. It turns out that the 5 mg CNFs sprayed on the MXene aerogel sensor have the highest sensitivity, and the sensitivity of 10 mg CNF sprayed is the lowest since the isolation layer is too thick. Therefore, the 5 mg CNF isolation layer is selected for the MXene aerogel sensor.


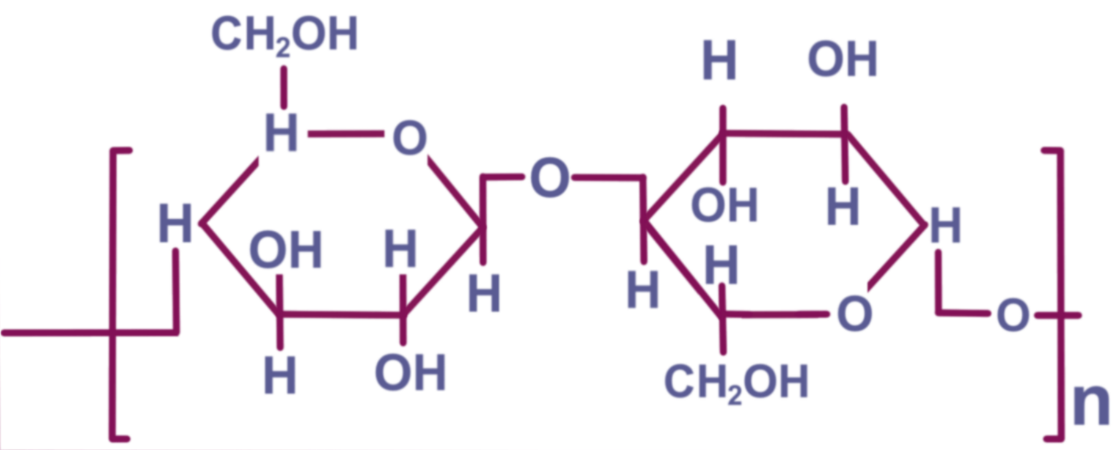


Figure S1. Structure of cellulose nanofibers (CNF, C_6_H_10_O_3_).


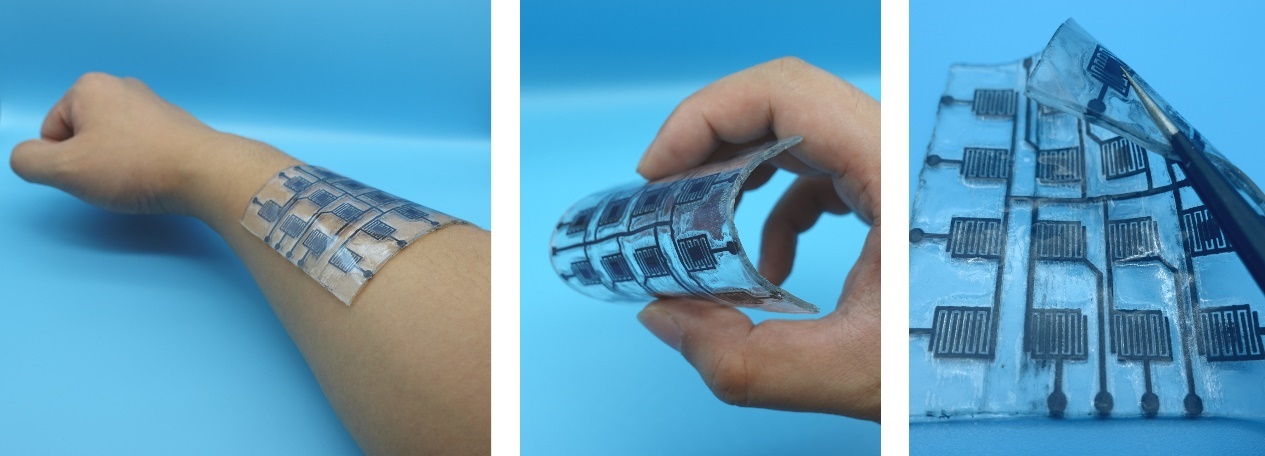


Figure S2. Photographs of MXene aerogel flexible pressure array sensor.


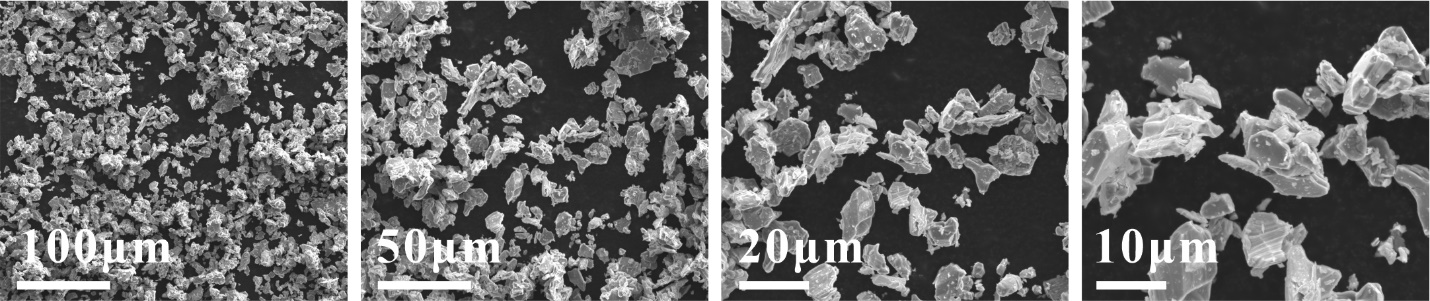


**Figure S3. SEM images of MAX phase (Ti_3_AlC_2_).**


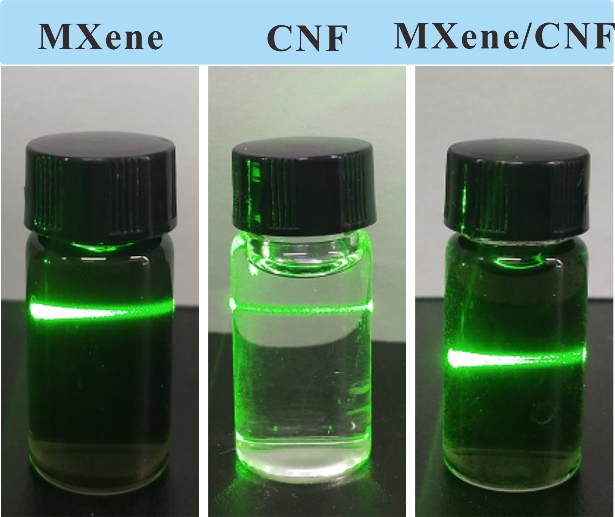


**Figure S4. Tyndall phenomenon of MXene, CNF, and MXene/CNF solutions.**


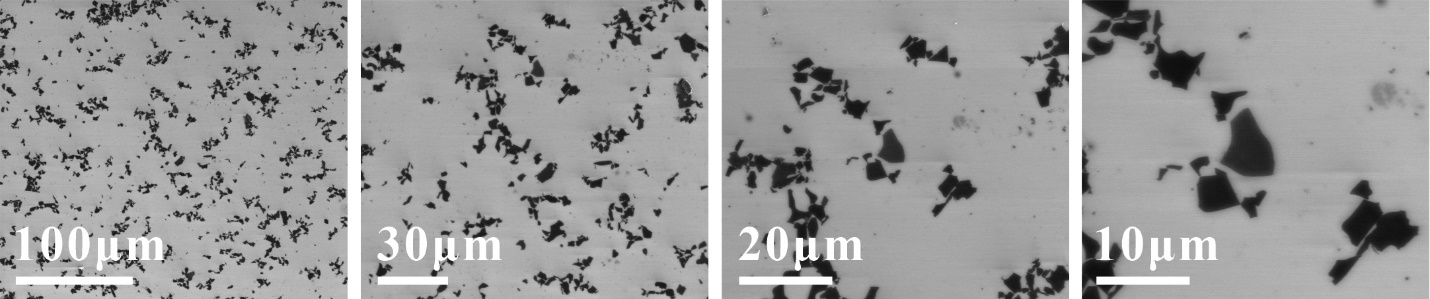


**Figure S5. SEM images of Ti_3_C_2_T_X_ MXene nanosheets.**


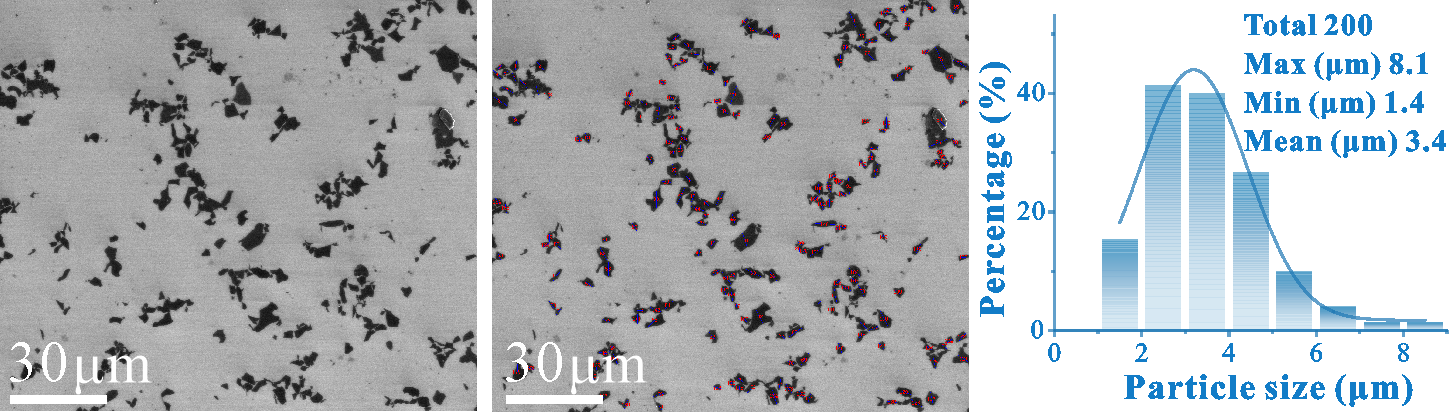


**Figure S6. SEM images of Ti_3_C_2_T_X_ MXene nanosheets and corresponding statistics of sheet size distribution.**


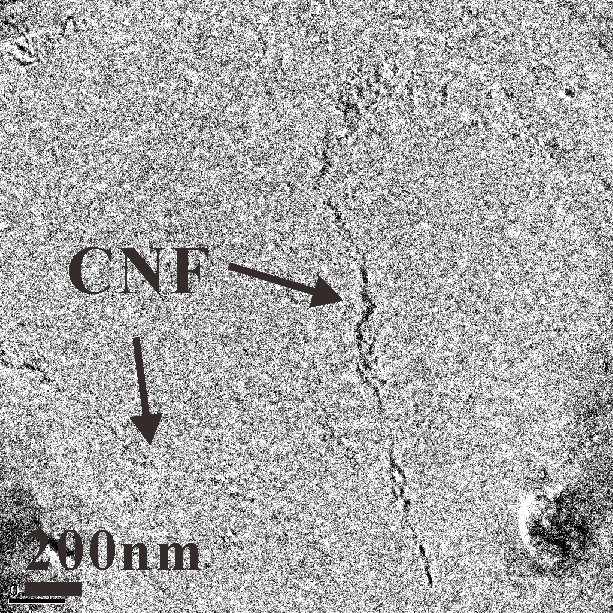


Figure S7. TEM image of cellulose nanofibers (CNF, C_6_H_10_O_3_).


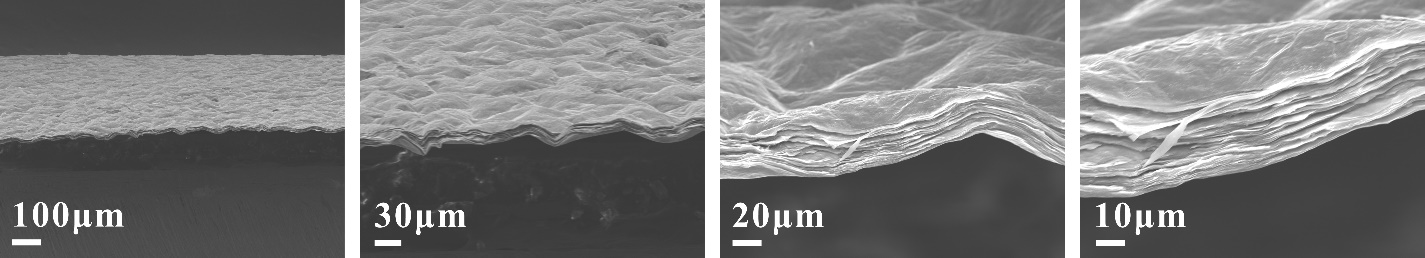


**Figure S8. SEM images of MXene/CNF film.**


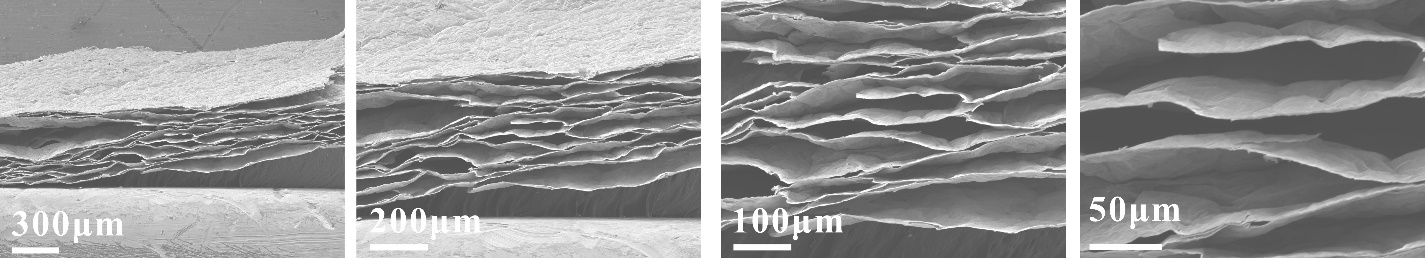


**Figure S9. SEM images of MXene/CNF aerogel.**


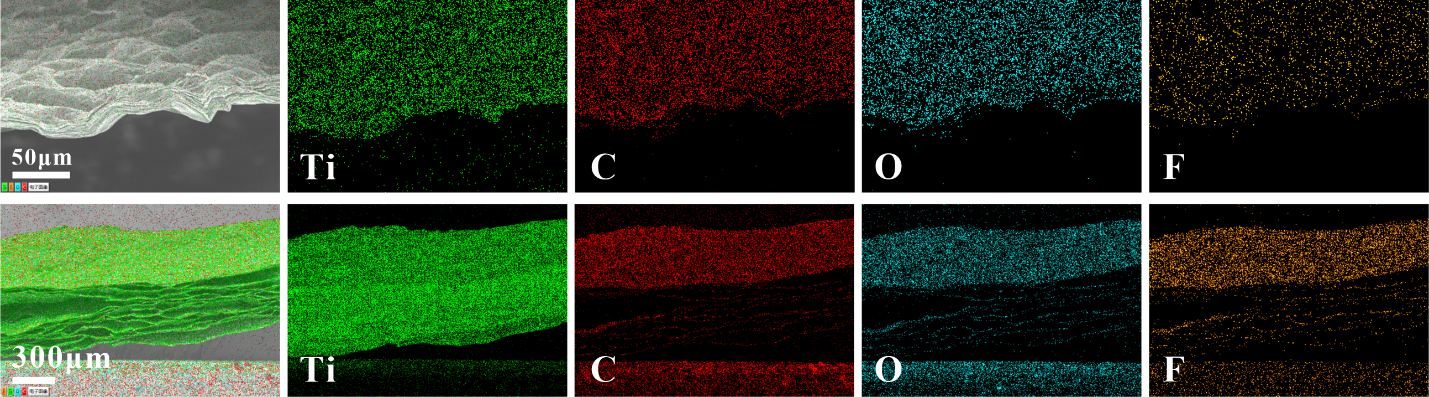


**Figure S10.** **Cross-sectional SEM images of MXene/CNF film (upper) and aerogel (lower), and the corresponding elemental maps.**


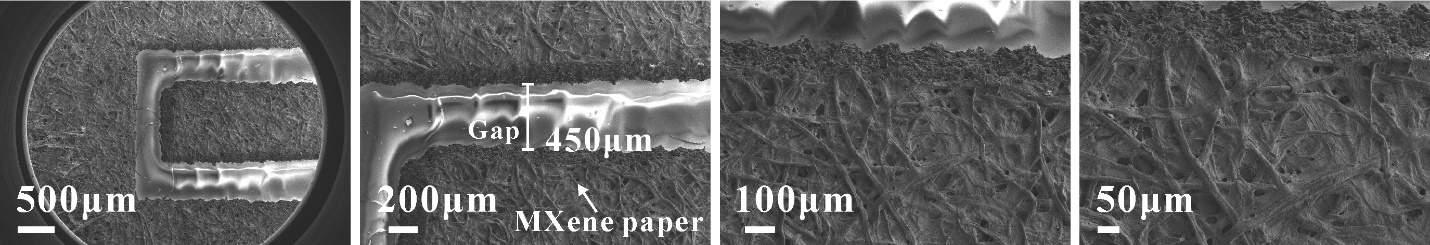


**Figure S11.** **SEM images of MXene paper interdigital electrodes.**


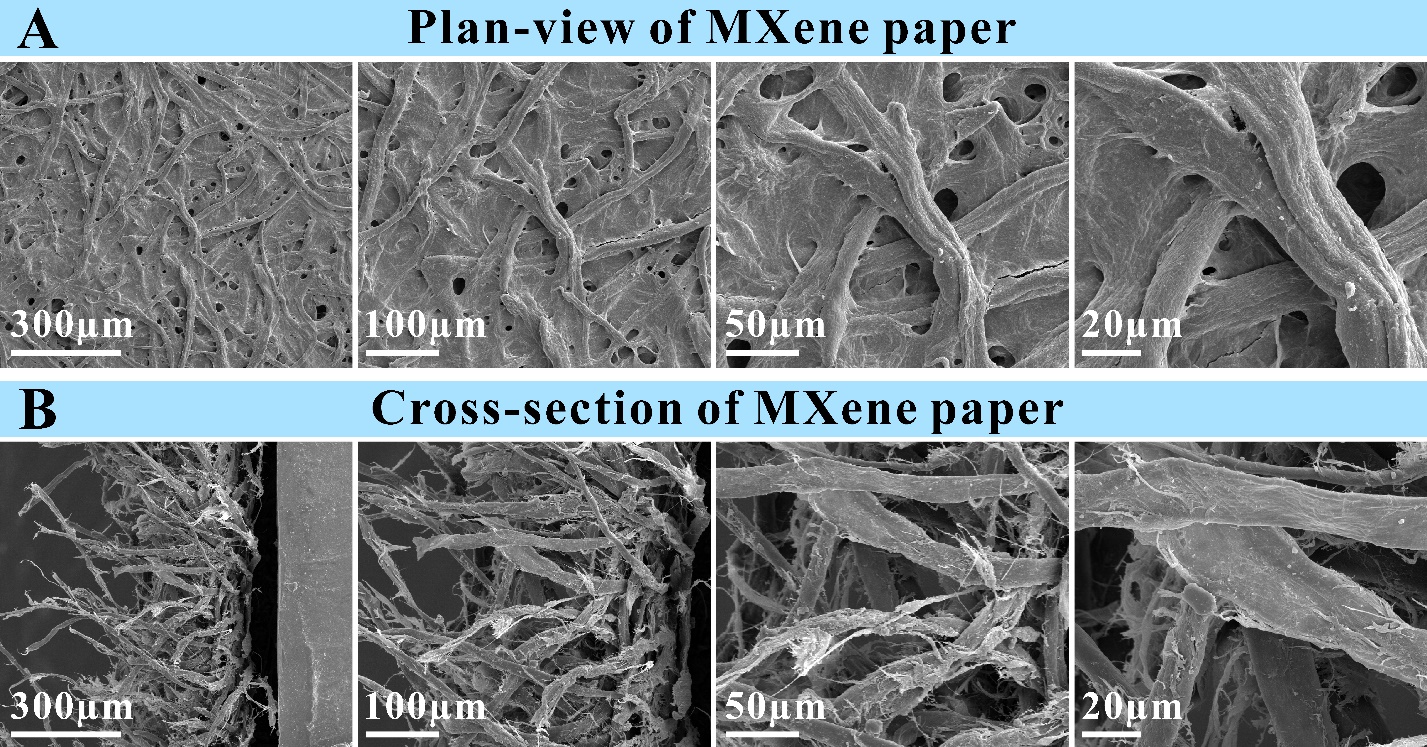


Figure S12. (A) Plan-view and (B) cross-sectional SEM images of MXene paper.


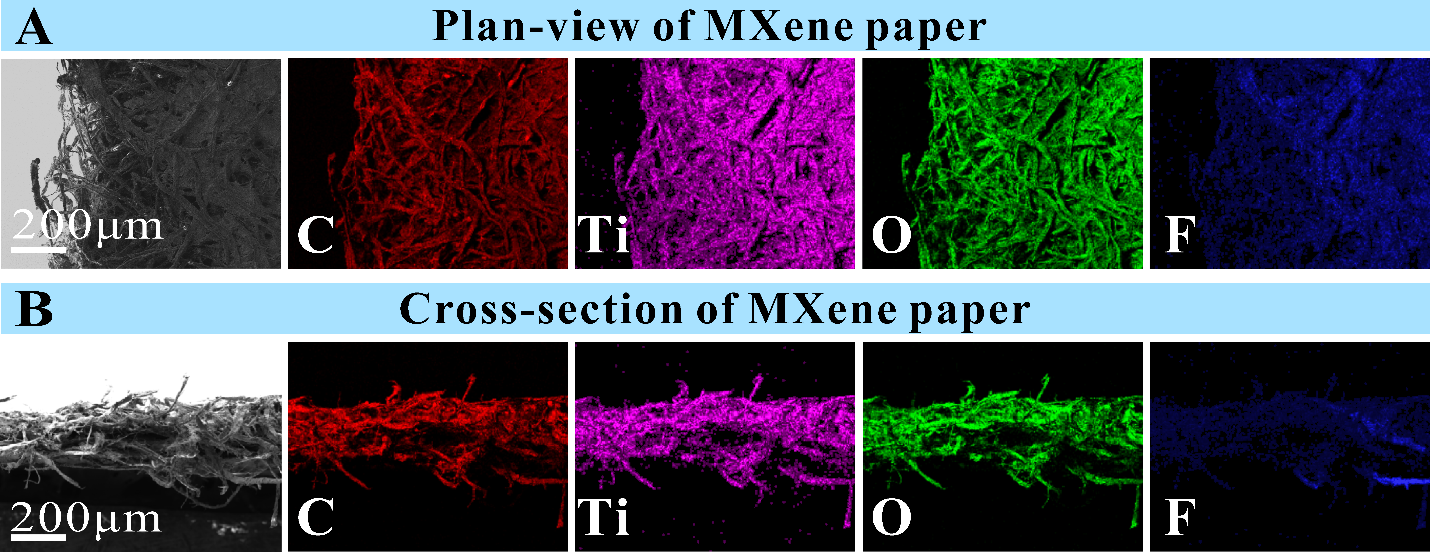


Figure S13. (A) Plan-view and (B) cross-sectional SEM images and corresponding EDS elemental maps of MXene paper.


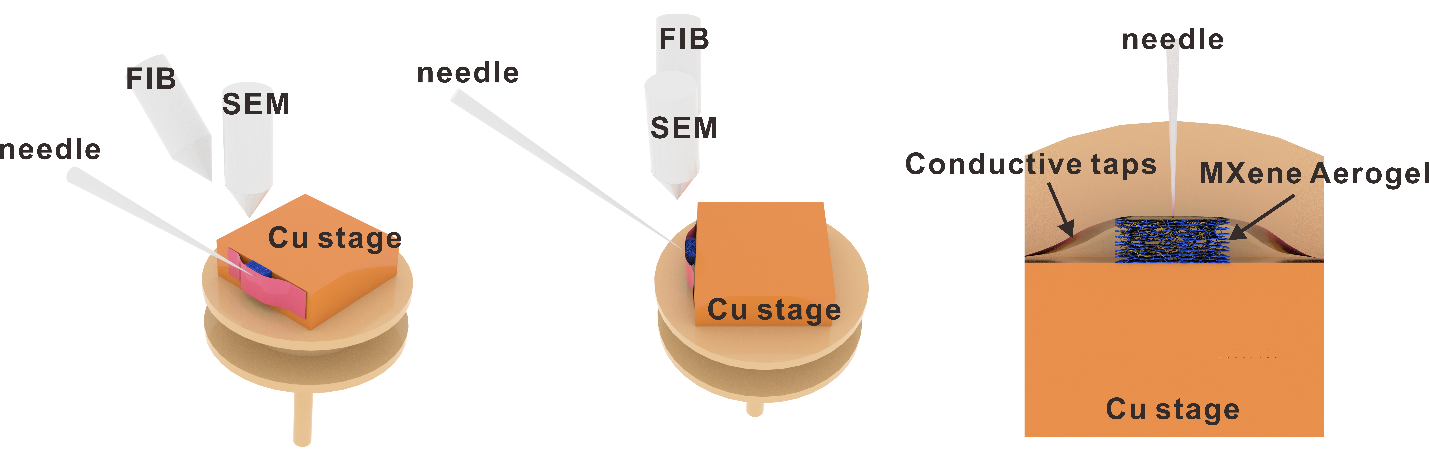


Figure S14. Schematic illustration of the *in-situ* mechanical test of MXene aerogel in FIB-SEM.


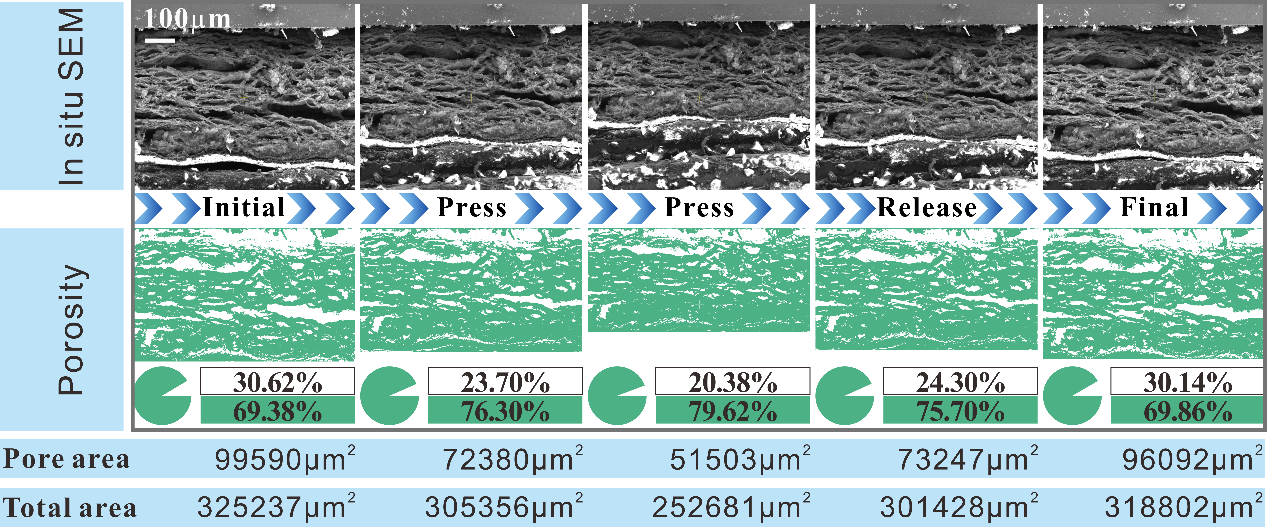


Figure S15. *In-situ* SEM images and corresponding porosity changes during dynamic press and release processes.


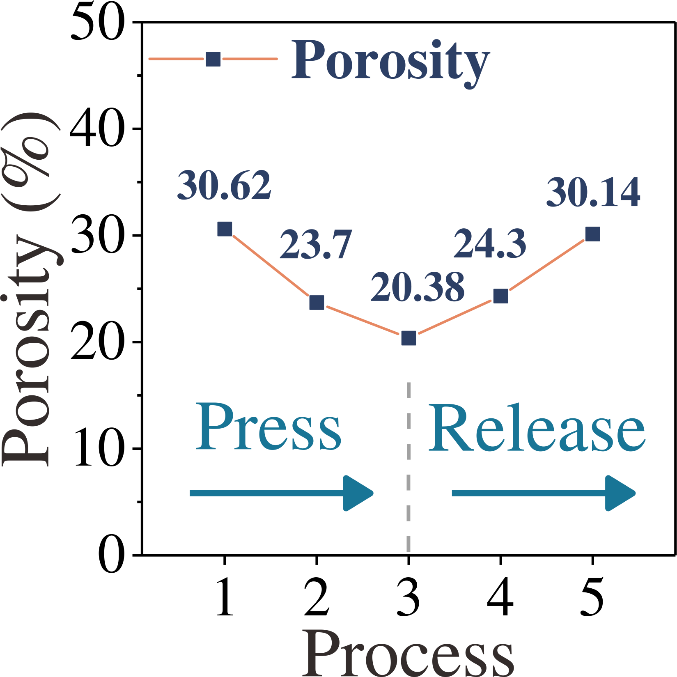


**Figure S16. The porosity changes of the MXene aerogel during dynamic press and release processes.**


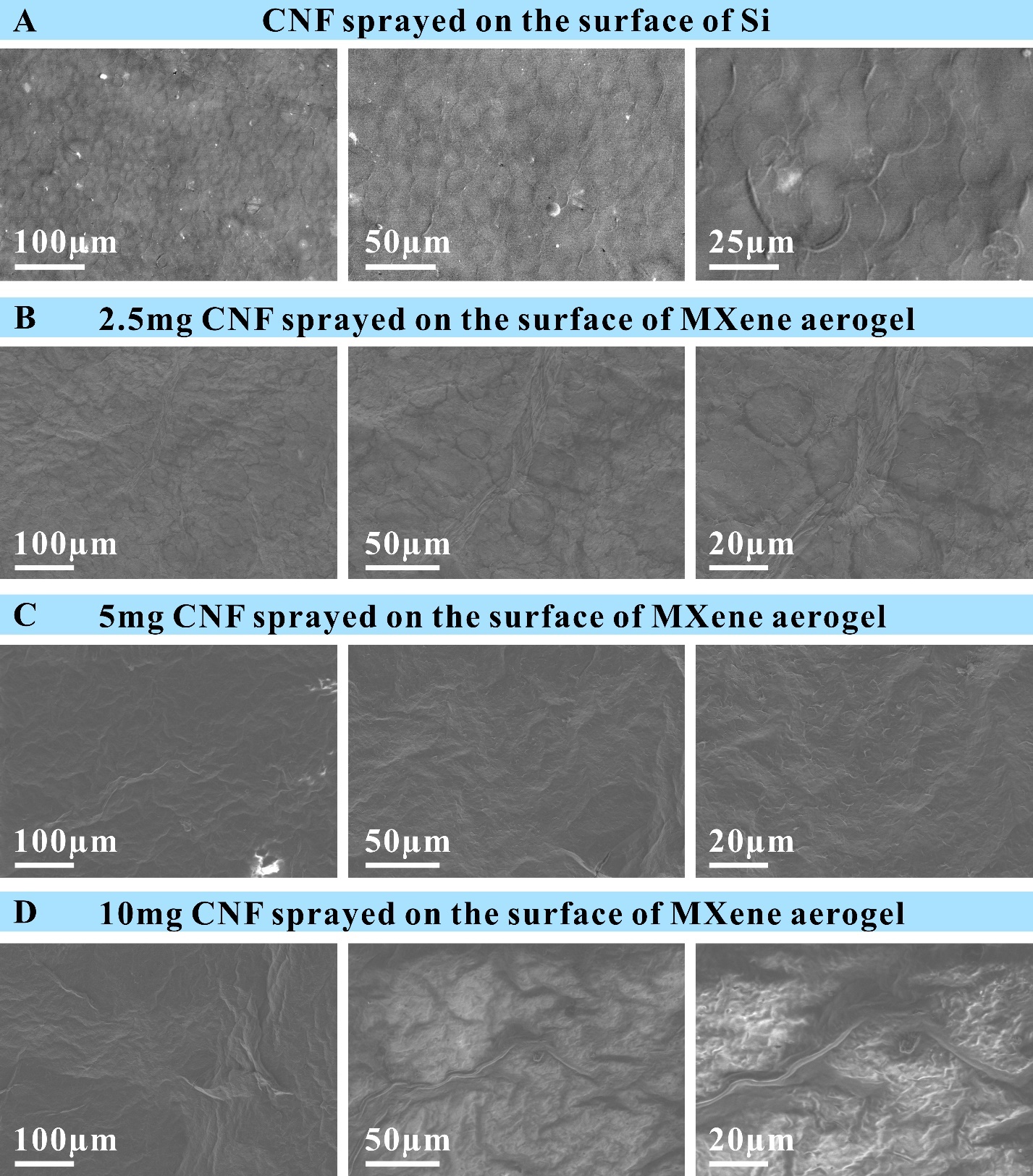


Figure S17. SEM images of sprayed CNF isolation layer. (A) CNF sprayed on Si surface. (B) 2.5 mg CNF, (C) 5 mg CNF, and (D) 10 mg CNF were sprayed on the surface of MXene aerogel.


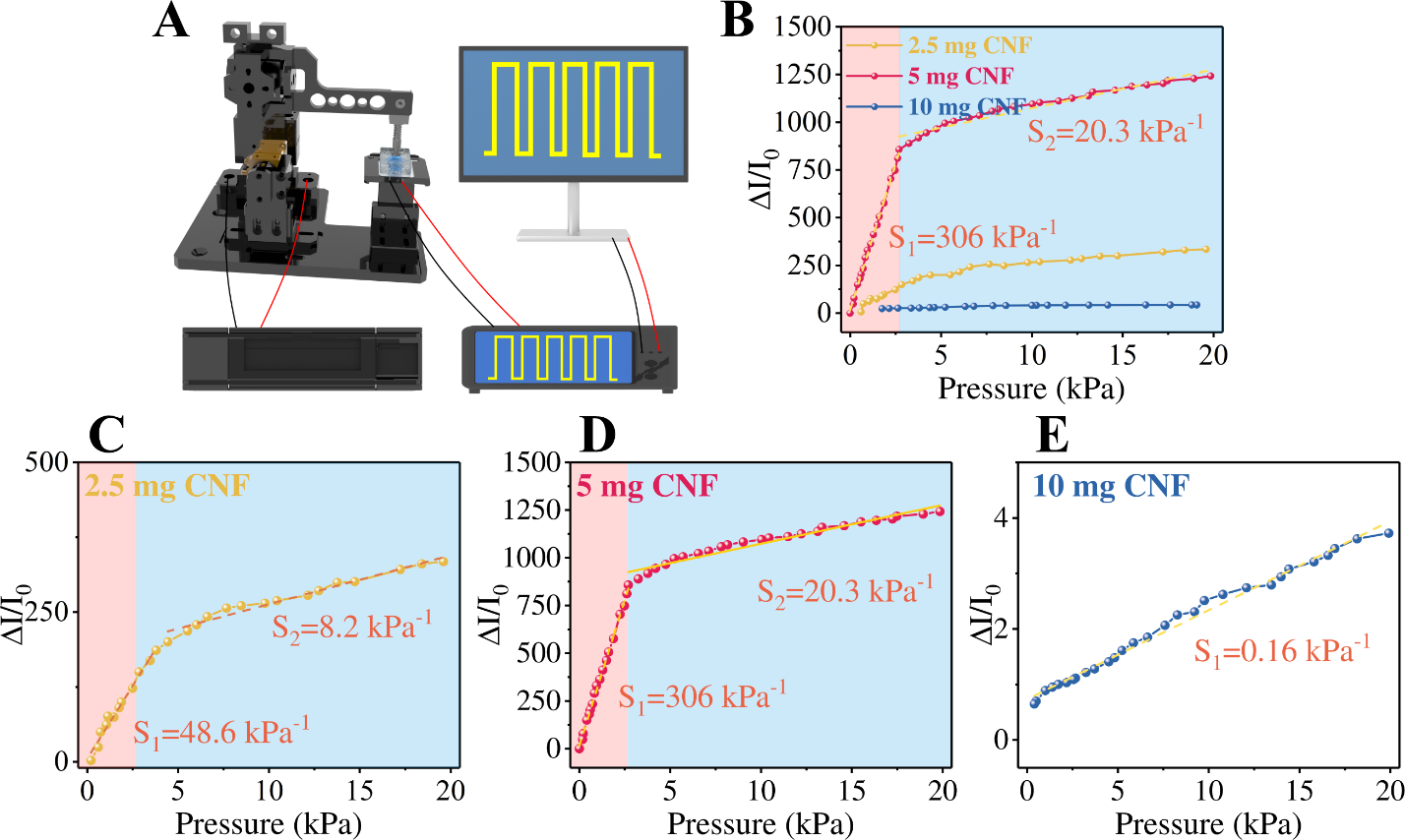


Figure S18. Sensitivity of MXene aerogel sensor with different thicknesses of CNF isolation layer. (A) Schematic diagram of the high-precision pressure-current test system. Sensitivity plots of (B) Total. (C) 2.5 mg CNF. (D) 5 mg CNF. (E) 10 mg CNF.


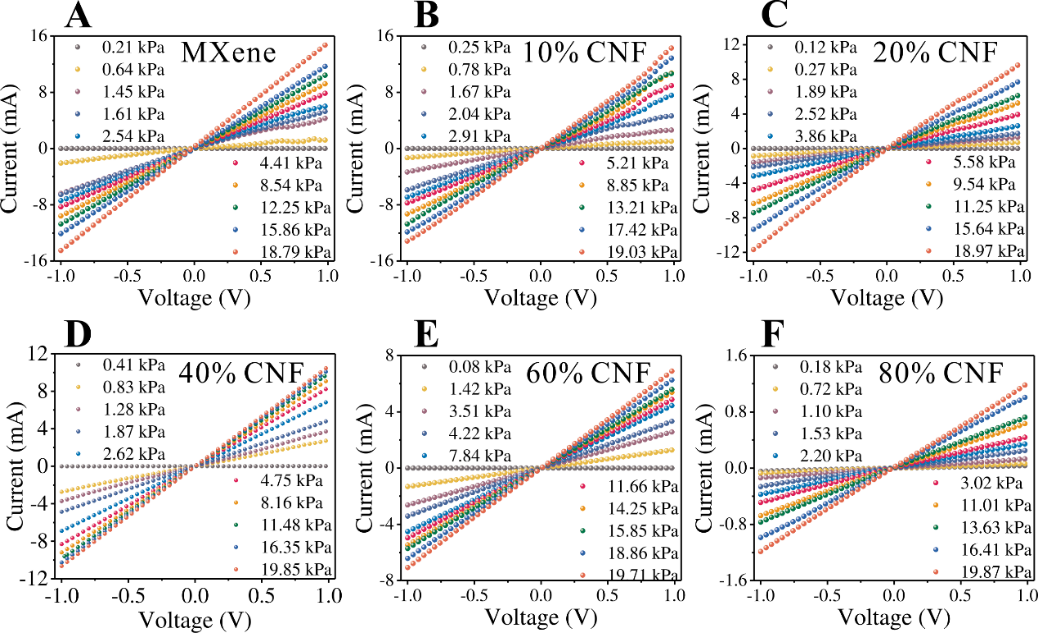


Figure S19. Ohmic contact characteristics of MXene aerogel sensor. I–V curves of MXene aerogel sensor with different CNF contents under various pressures (A) MXene, (B) 10 wt% CNF, (C) 20 wt% CNF, (D) 40 wt% CNF, (E) 60 wt% CNF, (F) 80 wt% CNF.


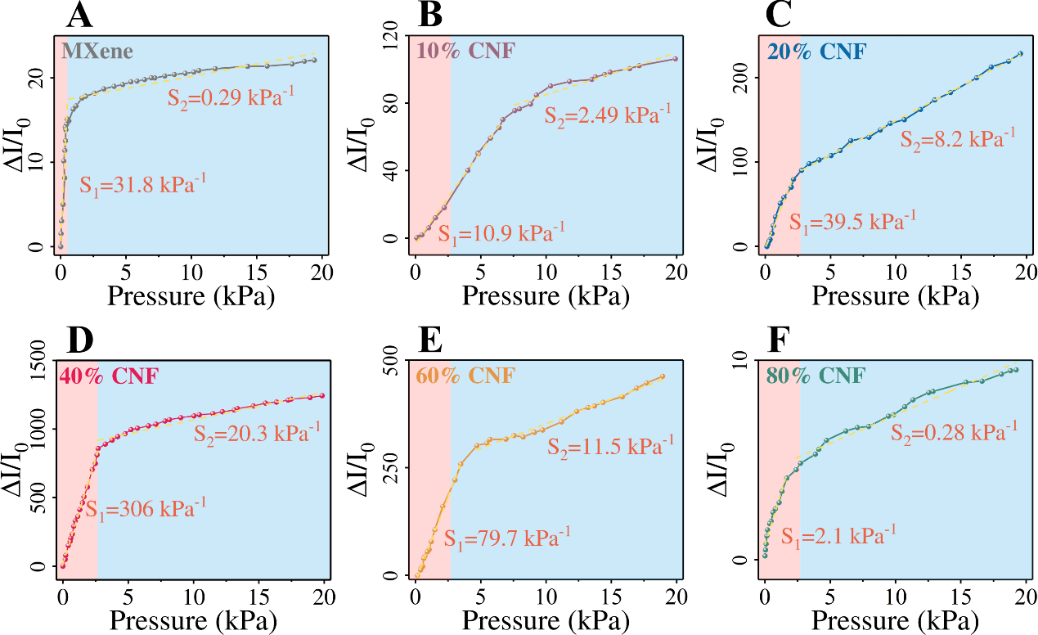


Figure S20. Sensitivity of MXene aerogel sensor with different CNF contents. (A) MXene aerogel. (B) 10 wt% CNF MXene aerogel. (C) 20 wt% CNF MXene aerogel. (D) 40 wt% CNF MXene aerogel. (E) 60 wt% CNF MXene aerogel. (F) 80 wt% CNF MXene aerogel.


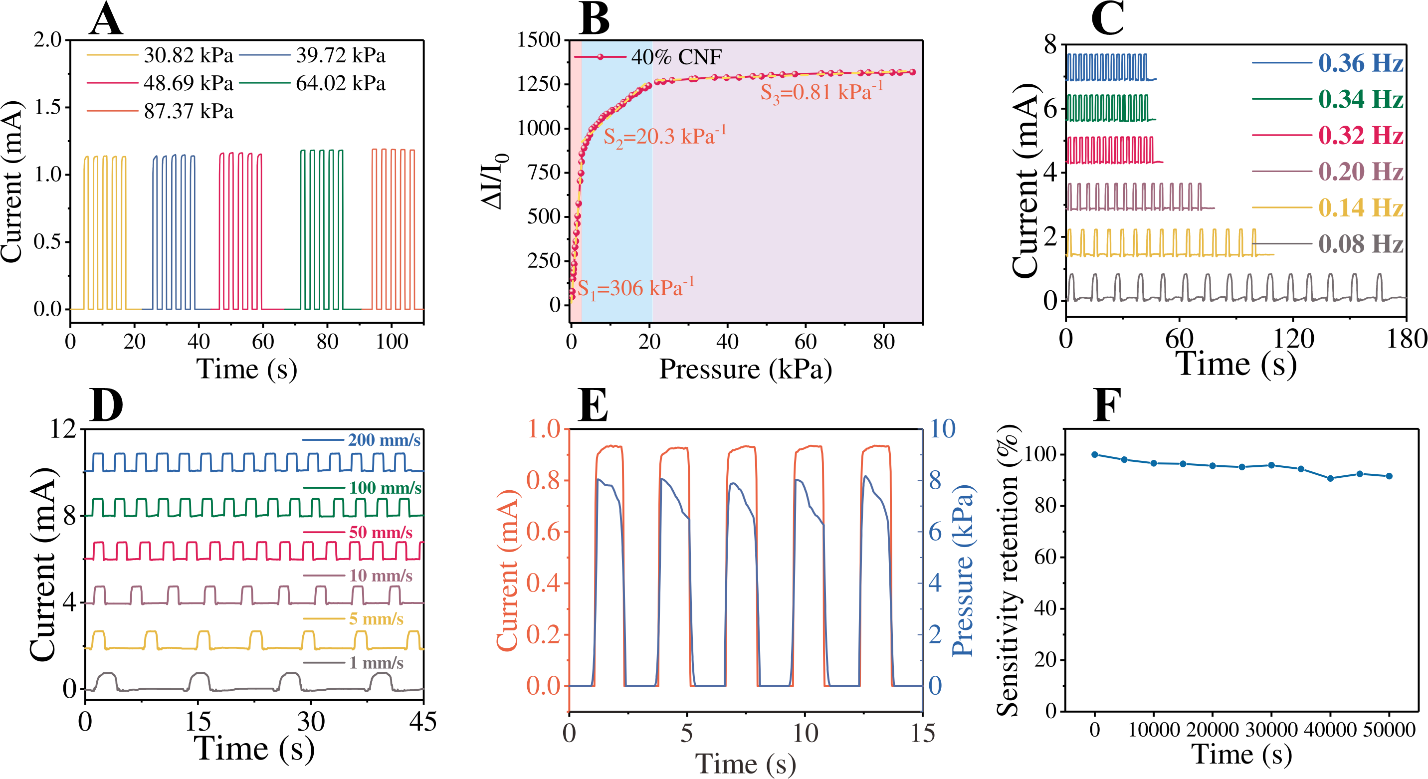


Figure S21. Pressure-sensing properties of MXene aerogel sensor. (A) I–T curves of 40 wt% CNF MXene aerogel sensor when the pressure is more than 20 kPa. (B) Sensitivity of 40 wt% CNF MXene aerogel sensor. (C) Frequency and (D) speed responses of MXene aerogel sensor at 4.75 kPa. (E) The P-T curve is in high agreement with the I-T curve at 8.16 kPa. (F) The sensitivity of the sensor remains over 90% of the initial value after 20,000 cycles.


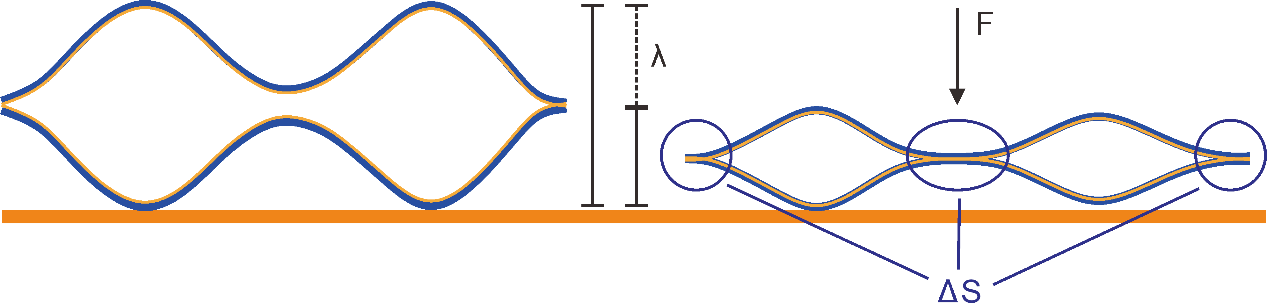


**Figure S22. Schematic diagram of the changing conduction channel.** The area change of the conduction channel (ΔS) when the strain is λ under the action of the force (F).


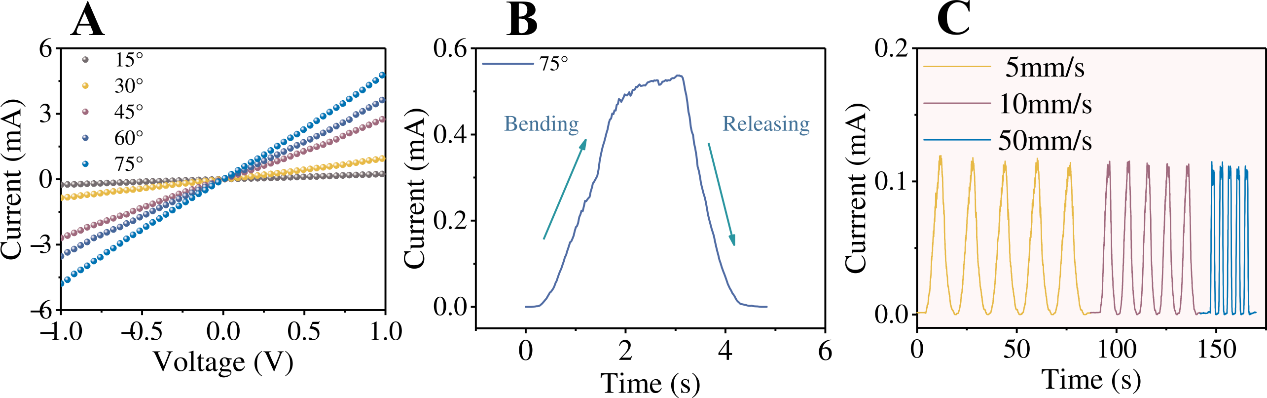


Figure S23. Tension-sensing properties of MXene aerogel sensor. (A) I–V curves of 40 wt% CNF MXene aerogel sensor at different bending angles. (B) One cycle of bending and releasing process of MXene aerogel sensor bent at 75°. (C) Speed response of MXene aerogel sensor with the bending angle of 30°.


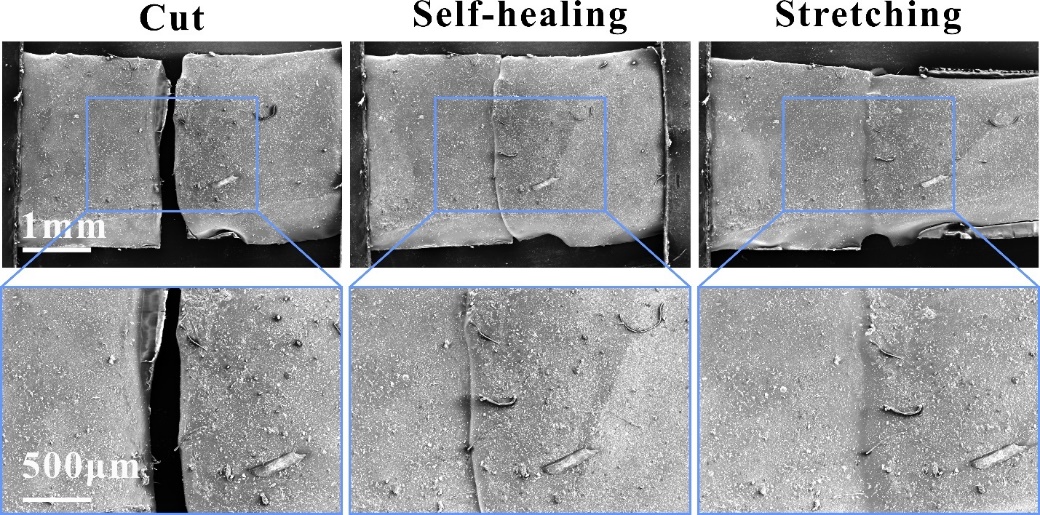


Figure S24. SEM images show the self-healing processes of PU including cut, self-healing, and stretching.


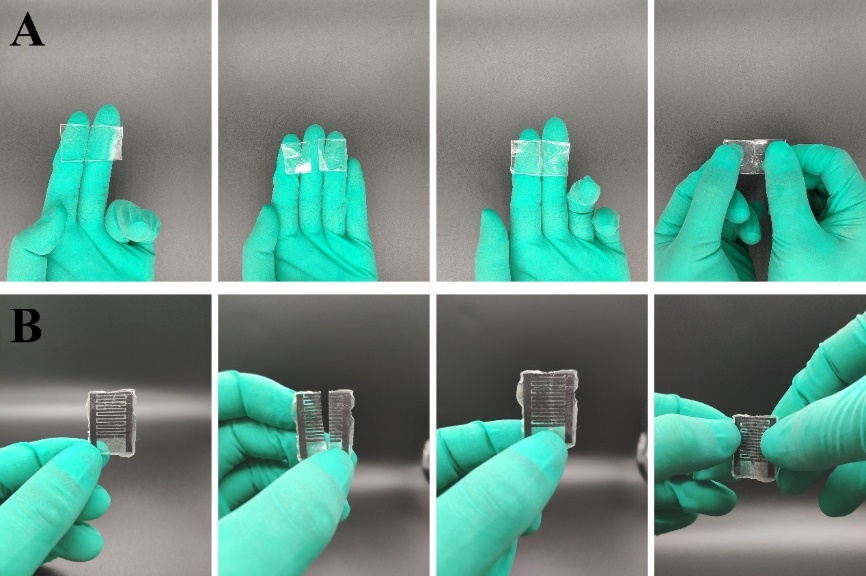


Figure S25. Photographs of self-healing characteristics of (A) PU and (B) interdigital electrodes.


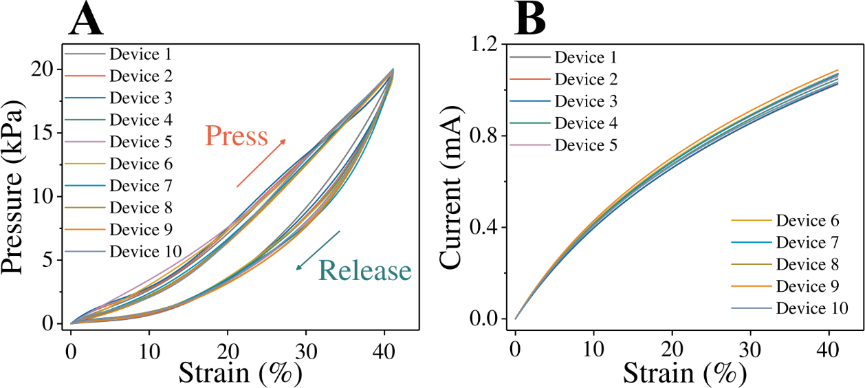


Figure S26. The reliability of MXene aerogel sensors. (A) The mechanical and (B) sensing performances of ten batches of MXene aerogel sensors.


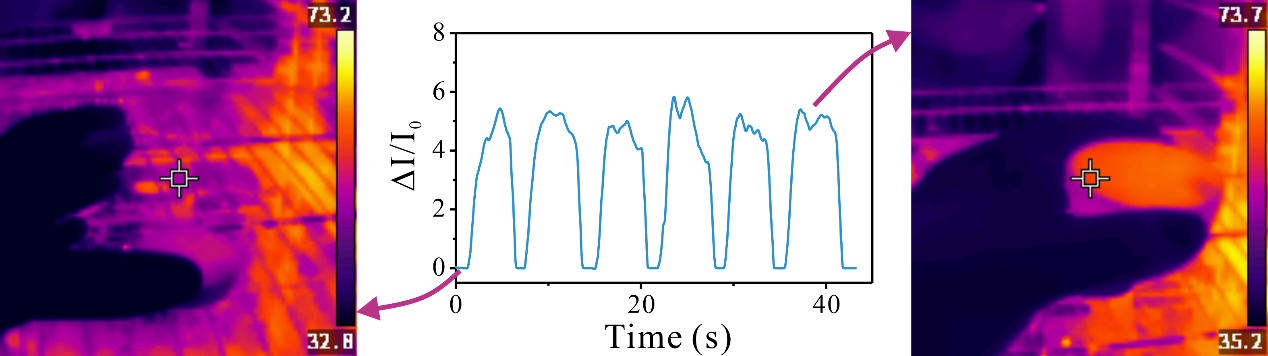


**Figure S27. The sensor attached to the hand can recognize the grabbing action of a hot water glass.**


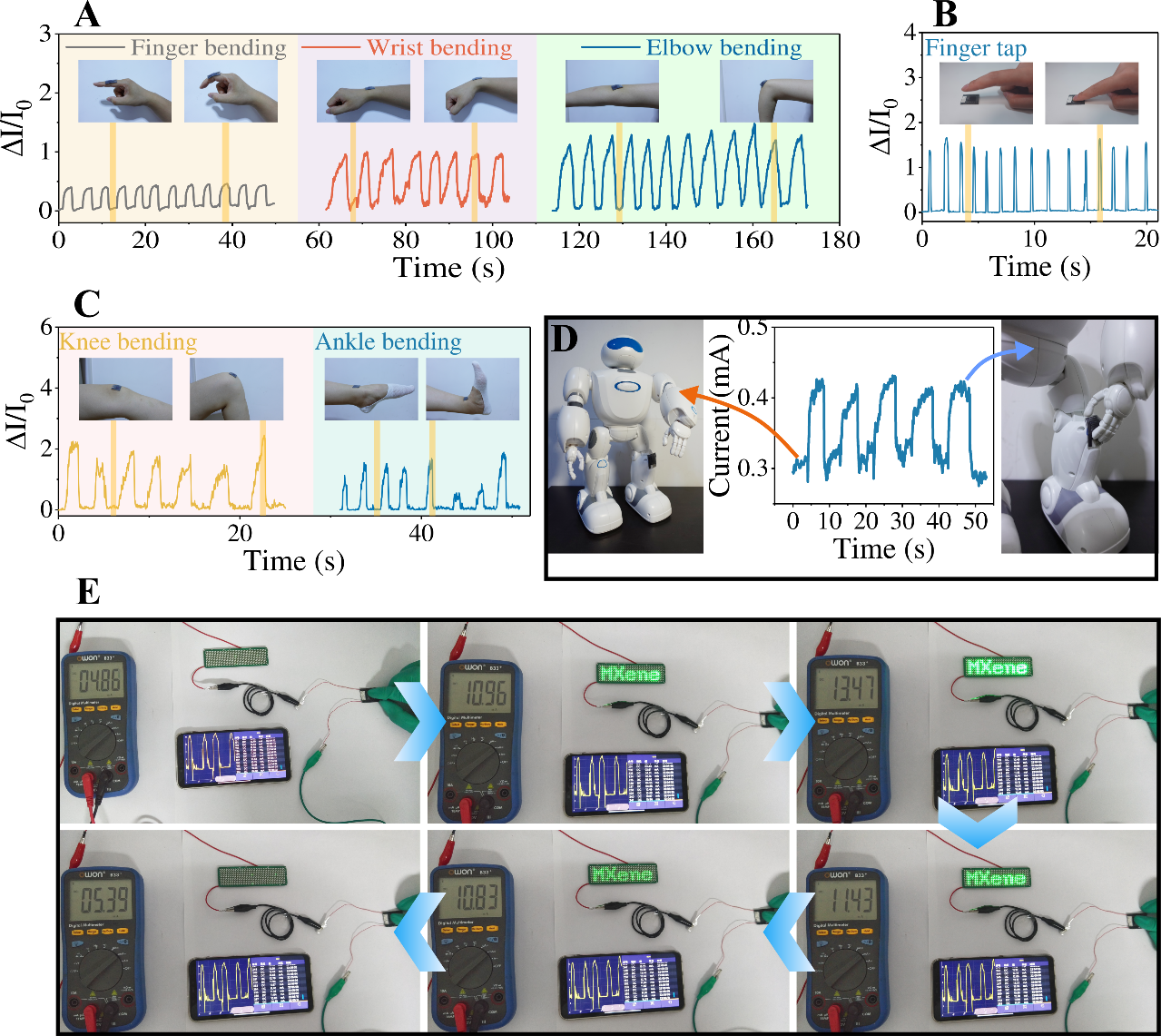


Figure S28. Practical applications of the multifunctional MXene aerogel flexible sensor. The current output of the sensor, corresponds to (A) finger bending, wrist bending, and elbow bending; (B) finger tap; (C) knee bending and ankle bending; (D) robot motion behavior detection. (E) The human-computer interaction interface for remote monitoring and controlling the brightness of LED light signs.


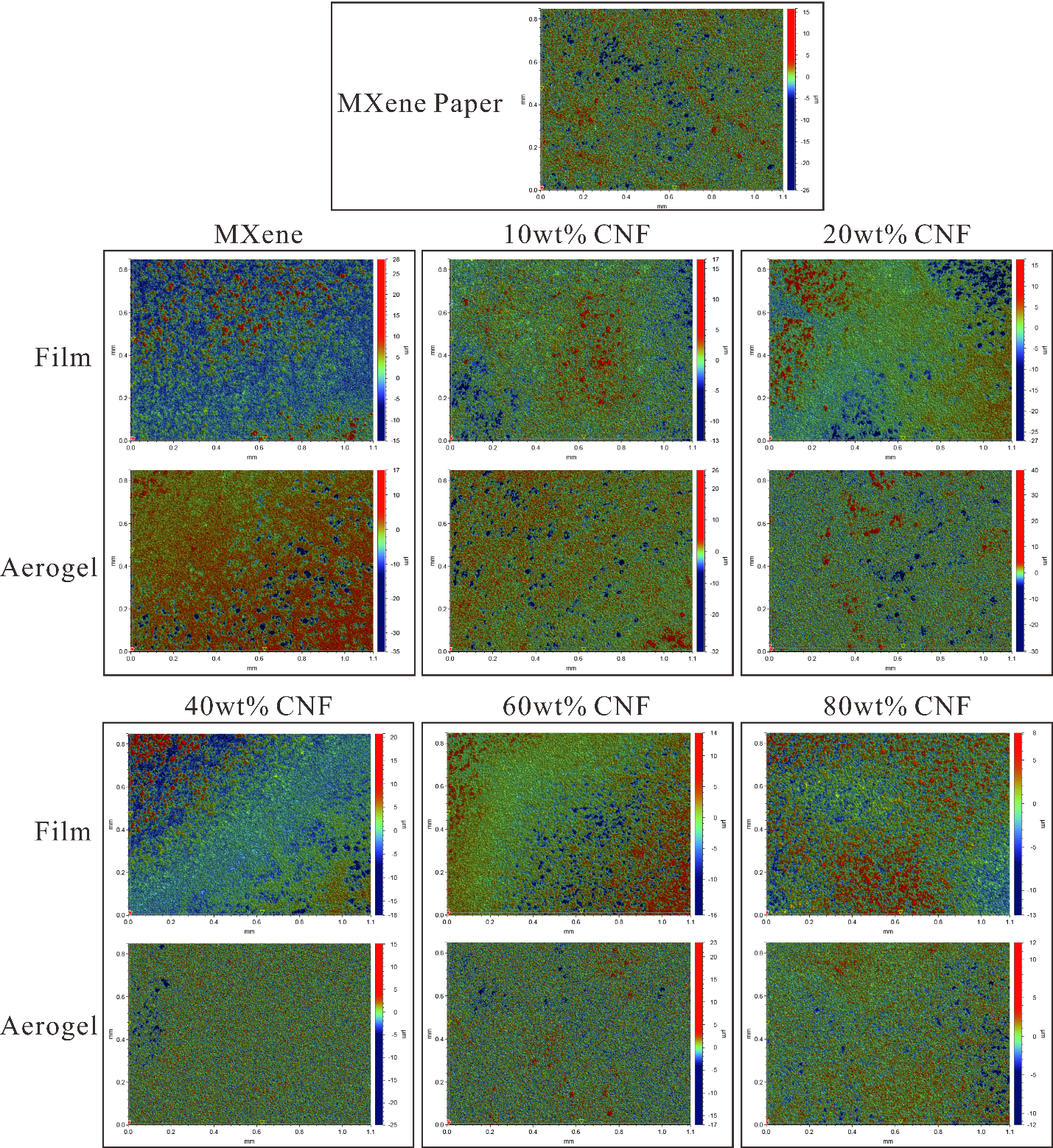


Figure S29. Surface profiles of MXene paper, MXene films, and aerogels with different CNF contents.


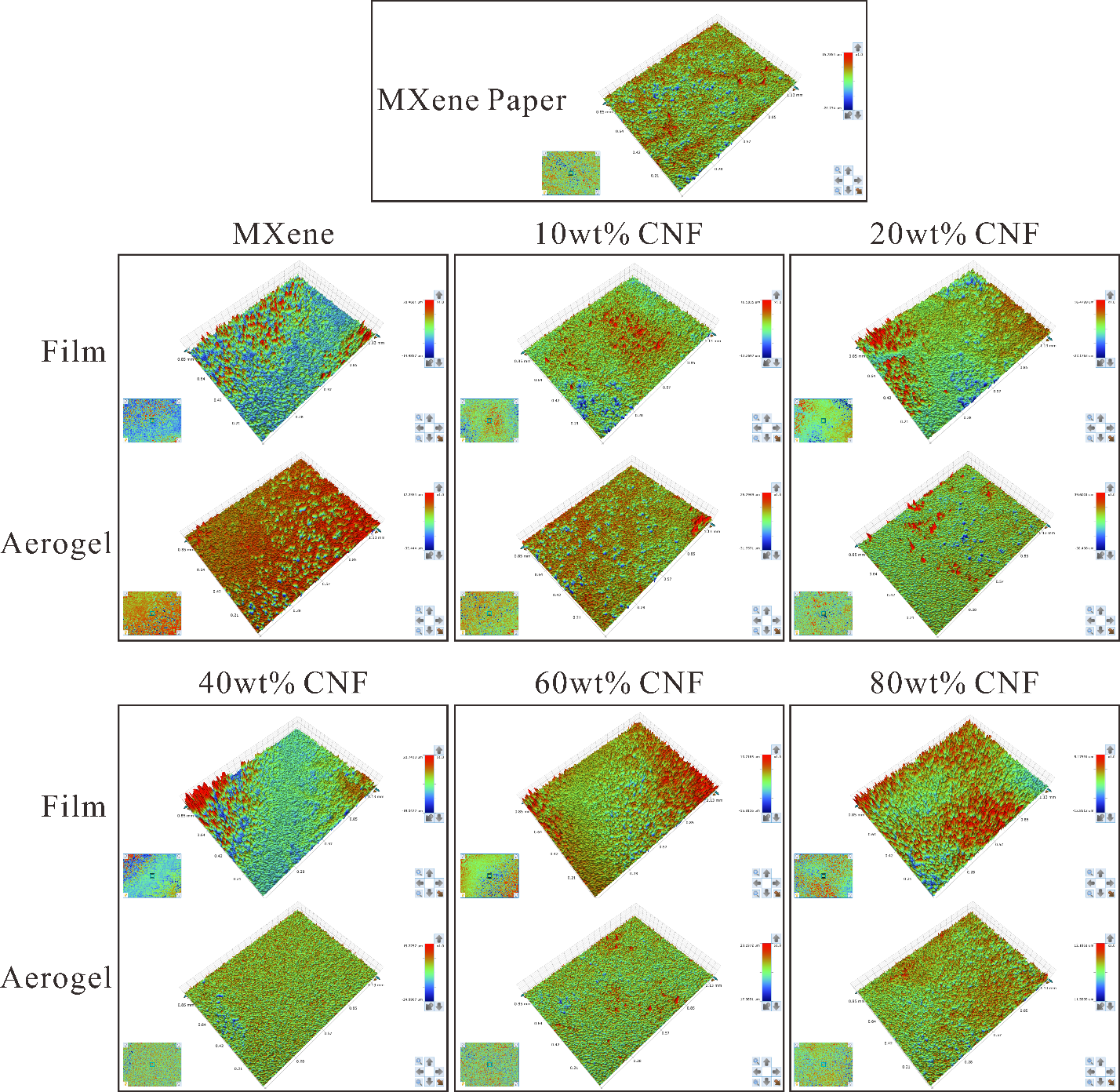


Figure S30. 3D morphologies of MXene paper, MXene films, and aerogels with different CNF contents.


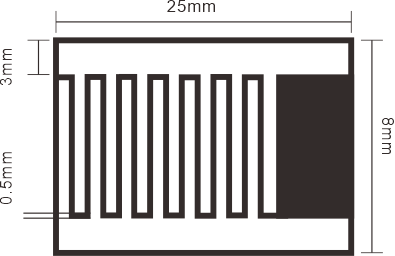


**Figure S31. Dimensions of interdigital electrodes.**

Table S1. Comparison of 3D MXene aerogel sensor and other sensors.

| Materials | Method | Production time | Sensitivity | Pressure range | Response time | Self-healing ability |
| --- | --- | --- | --- | --- | --- | --- |
| MXene/CNF  (Our work) | Gas foaming | < 1h | 306 kPa^-1^ | 2.3 Pa to 87.3 kPa | 35 ms | YES |
| Pt/PDMS (Nat. Mater.2012)[1] | Si template | > 12h | 11.45 | 5 Pa to 1.5 k Pa | 50 ms | NO |
| Graphene/PDMS (Sci. Adv. 2020)[2] | Composites | > 12h | 15.22 kPa^-1^ | 0.2 kPa to 40 kPa | 74 ms | NO |
| SWNTs/rGO/PDMS (Adv. Mater. 2018)[3] | Template, spraying | > 12h | 0.7 kPa^-1^ | < 25 kPa | 50 ms | NO |
| Carbonaceous Nanofibrous Aerogel (Adv. Mater.2016)[4] | Freeze-drying | > 48h | 1.02 kPa^-1^ | 10 Pa to  4.5 kPa | - | NO |
| MXene/rGO Aerogel (ACS Nano 2018)[5] | Freeze-drying | > 48h | 22.56 kPa^-1^ | 10 Pa to 3.5 kPa | 245 ms | NO |
| Graphene/Silicone (Adv. Mater. 2017)[6] | CVD, template | > 48h | 15.9 kPa^-1^ | < 60 kPa | 1.2 ms | NO |
| Black Phosphorus/MXene (Adv. Mater. 2021)[7] | Composites | > 12h | 77.61 kPa^-1^ | < 24.2 kPa | 10.9 ms | NO |

Table S2. Surface roughness of MXene paper, MXene films and MXene aerogels.

| **Parameters** | **Ra**  **Average roughness (μm)** | **Rp**  **Maximum peak height (μm)** | **Rv**  **Maximum valley depth (μm)** |
| --- | --- | --- | --- |
| MXene paper | 1.48 | 15.79 | -26.23 |
| MXene film | 2.41 | 28.46 | -14.99 |
| MXene aerogel | 2.46 | 17.26 | -35.44 |
| 10wt%CNF MXene/CNF film | 1.35 | 16.58 | -13.27 |
| 10wt%CNF MXene/CNF aerogel | 1.70 | 25.80 | -31.76 |
| 20wt%CNF MXene/CNF film | 2.27 | 16.48 | -27.18 |
| 20wt%CNF MXene/CNF aerogel | 3.14 | 39.60 | -30.44 |
| 40wt%CNF MXene/CNF film | 2.26 | 20.74 | -18.04 |
| 40wt%CNF MXene/CNF aerogel | 5.25 | 15.23 | -24.90 |
| 60wt%CNF MXene/CNF film | 1.35 | 13.72 | -15.81 |
| 60wt%CNF MXene/CNF aerogel | 2.20 | 23.20 | -17.09 |
| 80wt%CNF MXene/CNF film | 1.24 | 8.18 | -12.85 |
| 80wt%CNF MXene/CNF aerogel | 1.41 | 12.19 | -11.50 |

**References**

[1] C. Pang, G. Y. Lee, T. I. Kim *et al.*, A flexible and highly sensitive strain-gauge sensor using reversible interlocking of nanofibres, (in eng), Nat Mater*,* 2012;11:795-801.

[2] Y. Wang, H. Wu, L. Xu *et al.*, Hierarchically patterned self-powered sensors for multifunctional tactile sensing, (in eng), Sci Adv*,* 2020;6:eabb9083.

[3] G. Y. Bae, J. T. Han, G. Lee *et al.*, Pressure/Temperature Sensing Bimodal Electronic Skin with Stimulus Discriminability and Linear Sensitivity, (in eng), Adv Mater*,* 2018;30:e1803388.

[4] Y. Si, X. Wang, C. Yan *et al.*, Ultralight Biomass-Derived Carbonaceous Nanofibrous Aerogels with Superelasticity and High Pressure-Sensitivity, (in eng), Adv Mater*,* 2016;28:9512-9518.

[5] Y. Ma, Y. Yue, H. Zhang *et al.*, 3D Synergistical MXene/Reduced Graphene Oxide Aerogel for a Piezoresistive Sensor, (in eng), ACS Nano*,* 2018;12:3209-3216.

[6] N. Luo, Y. Huang, J. Liu *et al.*, Hollow-Structured Graphene-Silicone-Composite-Based Piezoresistive Sensors: Decoupled Property Tuning and Bending Reliability, (in eng), Adv Mater*,* 2017;29:

[7] Y. Zhang, L. Wang, L. Zhao *et al.*, Flexible Self-Powered Integrated Sensing System with 3D Periodic Ordered Black Phosphorus@MXene Thin-Films, (in eng), Adv Mater*,* 2021;33:e2007890.
